# The enemy of my enemy is my friend: native pine marten recovery reverses the decline of the red squirrel by suppressing grey squirrel populations

**DOI:** 10.1098/rspb.2017.2603

**Published:** 2018-03-07

**Authors:** Emma Sheehy, Chris Sutherland, Catherine O'Reilly, Xavier Lambin

**Affiliations:** 1 School of Biological Sciences, University of Aberdeen, Zoology building, Tillydrone Avenue, Aberdeen AB24 2TZ, UK; 2 Department of Environmental Conservation, University of Massachusetts-Amherst, Amherst, MA, USA; 3 Department of Science, Waterford Institute of Technology, Waterford, Ireland

**Keywords:** occupancy modelling, spatial capture–recapture, apparent competition, predator-mediated competition, pest-regulating ecosystem service, species interactions

## Abstract

Shared enemies may instigate or modify competitive interactions between species. The dis-equilibrium caused by non-native species introductions has revealed that the outcome of such indirect interactions can often be dramatic. However, studies of enemy-mediated competition mostly consider the impact of a single enemy, despite species being embedded in complex networks of interactions. Here, we demonstrate that native red and invasive grey squirrels in Britain, two terrestrial species linked by resource and disease-mediated apparent competition, are also now linked by a second enemy-mediated relationship involving a shared native predator recovering from historical persecution, the European pine marten. Through combining spatial capture–recapture techniques to estimate pine marten density, and squirrel site-occupancy data, we find that the impact of exposure to predation is highly asymmetrical, with non-native grey squirrel occupancy strongly negatively affected by exposure to pine martens. By contrast, exposure to pine marten predation has an indirect positive effect on red squirrel populations. Pine marten predation thus reverses the well-documented outcome of resource and apparent competition between red and grey squirrels.

## 1. Introduction

Understanding the mechanisms through which species interact and the consequences of perturbations to those interactions is a fundamental goal of ecology. Species interactions are typically not pairwise, but rather are embedded in complex interaction networks. For instance, two-species interactions may be mediated, in part or wholly, by the presence of a third species, as in predator- or pathogen-mediated apparent competition [1]. The potential outcomes of such indirect interactions include complete competitor exclusion and fugitive coexistence where inferior competitors thrive temporarily in the absence of a shared enemy [2]. Which outcomes emerge depends on the nature of trophic interactions, including how the natural enemy affects, and responds to, the competing prey/host species, and on the ability of prey to occupy refuges that are temporarily or permanently unoccupied by their enemies.

The introduction of non-native species has provided some dramatic examples of indirect interactions leading to species extirpation. For example, pathogen-mediated apparent competition between invasive grey squirrels, *Sciurus carolinensis*, and native red squirrels, *Sciurus vulgaris*, in Britain where the process of replacement via exploitative competition is vastly expedited in the presence of squirrelpox virus (SQPV), a disease which is usually lethal to red squirrels but asymptomatic in grey squirrels which act as reservoir [3].

The occurrence of indirect interactions makes predicting the consequences of novel species interactions on biological networks challenging, especially in an applied context. This is illustrated by the many failures to achieve biological control of target invasive species through the introduction of non-native generalist predators which have resulted in unintended, often disastrous, direct and indirect impacts on native species ill-adapted to coexist with a novel predator [4]. Whether such dramatic impacts will emerge following the reinsertion of native predators on natural food webs which have been invaded by non-native prey is unknown.

Although in decline globally, hitherto persecuted native predator populations are recovering in Europe in response to large-scale conservation initiatives [5]. Predator recoveries take place in often profoundly modified landscapes used by native prey species with which they share a coevolutionary history, and by multiple non-native species that have invaded in their absence. While the eventual outcome of this modicum of ecosystem restoration is uncertain, a broad prediction is that the outcome of novel interactions between recovering native predators and non-native prey are more likely to lead to non-native species extirpation than interactions involving native prey species. More refined predictions are hampered by the contextual network of interactions such that so-called ‘contingent' rather than general theories are required to characterize circumstances where both direct and enemy-mediated interactions between species occur [1]. Specifically, predicting whether native predator recovery will lead to the extirpation of some non-native species or their fugitive coexistence with native guilds in a given context is, as yet, beyond the predictive ability of community ecology.

Despite the obvious relevance of indirect interactions for species and ecosystem conservation in the face of ever-increasing invasion pressure, empirically demonstrating the influence of enemy-mediated interactions is challenging, especially when dealing with low density and difficult to study mammalian predators. Performing manipulative experiments at relevant spatial scales is particularly difficult, and consequently, the evidence is often correlative. Yet, the standard of evidence required to shape policy is high, especially when some of the species involved are at the centre of conflicts. For instance, the controversy as to whether the dingo, *Canis dingo*, reduces the abundance or occupancy of introduced red foxes, *Vulpes vulpes*, and feral cats, *Felis catus*, and hence benefits endangered marsupials in the vulnerable prey size range in Australia, hinges on the need to account for imperfect detectability rather than using uncorrected indices of abundance [6].

Here, we investigate how the recovery of a native predator, the European pine marten, *Martes martes*, influences native red and non-native grey squirrels in Britain, two species that interact through exploitative competition and a form of disease-mediated apparent competition [3,7]. We ask whether these two species are linked by a second enemy-mediated relationship involving a shared predator, recovering from historical persecution, and we expect that the impact of the predator may be asymmetrical, due to a lack of similar predators in the invasive species' native range [8]. Sheehy & Lawton [9] reported a strong positive correlation between sightings of pine marten and red squirrel, and a negative correlation between pine marten and grey squirrel detection rates. While this evidence has profound implications both in the conservation of native red squirrels and the management of grey squirrels, the study focused on pairwise correlations of species incidences, which precludes investigation of the potential for interacting relationships between native and non-native species. Moreover, Sheehy & Lawton [9] concluded that pine marten density, rather than presence alone, may influence grey squirrels, although estimates of marten density are mostly lacking. Here, we tested the hitherto untested hypothesis that predator density is mediating competition between a non-native and native prey species using estimates of red and grey squirrel occupancy, and pine marten density and space use.

## 2. Material and methods

### (a) Survey design

Our survey took place between January and May 2016 in three regions of Scotland that differed in time since pine marten recolonization. Northernmost is the Highlands (HI: −4.7 W, 57.3 N around 150 km^2^), which was recolonized more than 45 years ago [10] and is beyond the current invasion range of the grey squirrel. Central Scotland (CS: −4.4 W, 56.1 N, around 900 km^2^), 100 km south of the HI region, was recolonized by pine martens approximately 8–14 years ago, and grey squirrels have been present since at least 1945 [11]. The Scottish Borders (BO: −3.2 W, 55.7 N, around 600 km^2^), 65 km southeast of the CS region, is in the early stage of pine marten recolonization (oldest contemporary record is less than 5 years old) and grey squirrels have been present since at least 1980 [12]. Grey squirrel control is ongoing in CS and BO: on average, 14 and 252 grey squirrels per year have been removed by culling since 2014, respectively (Scottish Wildlife Trust 2017, unpublished data). This take represents a small fraction of the standing grey squirrel population (0.2 and 5.1 individuals (ind) km^−2^ in CS and BO, respectively) when compared to reported densities of greater than 100 and 50 grey squirrels per km^2^ in broadleaf and coniferous habitat, respectively [13]. Moreover, the SQPV is endemic in BO [14] and was first detected in CS in 2017 (Scottish Wildlife Trust 2017, unpublished data).

In CS and BO, we used stratified random sampling to select 10 and seven 2 × 2 km grid cells, respectively, giving preference to those containing greater than 30% ‘broadleaved/mixed mainly broadleaved' habitat (electronic supplementary material, table S1). This focused the study in areas where grey squirrels were expected to have a competitive advantage over red squirrels [13] and, therefore, where species responses were not limited by habitat quality or availability. Each grid cell contained a minimum of approximately 70 hectares (ha) of suitable squirrel habitat, i.e. mature woodland (min = 68 ha, max = 400 ha, mean = 182 ha; electronic supplementary material, table S1) and between five and 21 sampling locations (hereafter ‘sites') (mean = 11.63) where multi-species feeders were permanently deployed a mean within-grid distance of 325 m apart (min = 267, max = 391). Mean home ranges of red and grey squirrels in these habitats are less than 5 ha [15,16], and hence each sampling site within a grid cell was considered independent at the scale of the squirrel species. In HI, where grey squirrels are absent, two grid cells were selected opportunistically based on site accessibility.

### (b) Multi-species sampling

Multi-species feeders were used to detect red and grey squirrels and pine martens. Detectors (15 × 15 × 15 cm wooden boxes with a wire mesh front and a liftable lid) were attached to trees at a height of 1.5 m at 223 sampling locations and were baited with a mixture of nuts and seeds that are attractive to martens and squirrels. On the underside of each lid, three 2 cm × 0.5 cm glue strips made from Big Cheese® glue traps were fixed to a pressure sensitive double-sided adhesive tab (3M™). To access the bait, squirrels and martens must climb a tree and lift the lid of the feeder with their head, resulting in hair samples being deposited on the glue strips, which can be used to identify squirrel species, and from DNA extracted from hair samples, individual martens. Each site was visited four times between January and May 2016 at approximately four-week intervals, with one additional visit to sites in the HI region in June. During each visit, hair samples were collected, new adhesives were deployed and feeders were re-baited.

Bushnell ‘Natureview' (model 119739) trail cameras were rotated between sites and visits throughout the study period such that during any sampling interval, a subset of sites (*n* = 19) had a camera and a feeder deployed. Cameras were positioned on an adjacent tree facing the feeder, and programmed to capture three images per trigger (at 0.6 s interval speed), followed by a 1 min trigger delay.

### (c) Genetic identification of marten individuals

Hair samples were dried and stored at −20°C to preserve DNA, and subsequently identified to species level, i.e. red squirrel, grey squirrel, pine marten, based on colour, shape and size using a dissection microscope at 40× magnification. Pine marten hair samples (greater than or equal to 10 where possible) were recovered from adhesives using 1–2 drops of xylene to soften the glue and transferred to 1.5 ml microfuge tubes using forceps that were heated and cooled between samples to avoid cross-contamination. Because more than one pine marten could visit a feeder between visits, hairs clumped together on a single adhesive strip were collected as unique samples.

To identify individual pine martens, DNA was extracted and real-time quantitative PCR assays for species and sex determination were carried out as described in [17]. Microsatellite amplification and allele scoring across six markers (Mel1, Ma2, Mvi1341, Gg7, Mar21 and Mar53) were performed as described in [18]. In samples where greater than two alleles per locus were observed (*n* = 4 samples), we assumed that this was a result of DNA amplification from more than one individual, and therefore samples were excluded from further analysis. Sample matching, calculation of allele frequencies and probabilities of identity were carried out using GENECAP [19]. For full details of microsatellite loci and primers used, see [18].

### (d) Spatial capture–recapture

Pine marten home ranges are larger than squirrels' and therefore, it is possible to detect hair from the same individual at multiple sites. The identification of unique individuals from hair samples generates individual spatial encounter histories (*y_ijk_*) documenting whether each observed individual (*i*) was observed at each site (*j*) during each occasion (*k*). Such data lend themselves naturally to analysis using spatial capture–recapture methods [20] that model encounter probabilities as an explicit function of the distance between site locations (*x_j_*) and individual activity centres (*s_i_*):
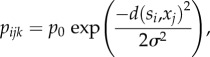
where *d*(*s_i_,x_j_*) is the distance between an individual's activity centre and a detector, *p*_0_ is the probability of encounter when that distance is 0, and *σ* is the spatial scale parameter that defines the distance over which the detection decreases. Here, to account for hypothesized variation in encounter probabilities, all combinations of sex- and region-specific detection parameters (*p*_0_ and *σ*) were compared.

Activity centres, which are not directly observed, are estimated using the spatial pattern of observations. The estimation of a spatial detection function that accounts for imperfect and spatially heterogeneous encounter rates allows inferences to be made about the total number of activity centres (individuals) within an area of interest, *S*, i.e. we estimate density. *S* is represented as the centre points of a discretized area that encompasses the sampling sites. This area must be large enough to contain the activity centres of all detected individuals; here a 4 km buffer was used which is larger than the radius of a pine marten home range (male and female home ranges 2.23 km^2^ and 1.49 km^2^, respectively) [21]. The resolution of the state space pixels must be fine enough to approximate continuous space but coarse enough to be computationally tractable; here the area was divided into 100 × 100 m pixels which is at least an order of magnitude smaller than a typical pine marten home range. Grid cells that were entirely water or non-forested habitat were removed assuming that density in these areas was 0. We tested for between-region differences in density by fitting models that assumed either constant density or region-specific density.

All combinations of encounter and density models were considered resulting in 32 candidate models. Models were ranked and weighted according to AIC [22]. In cases where no single model is overwhelmingly preferred, inference is based on model-averaged predictions using AIC weights. Models were fitted in R (R Core Team 2012) using the package oSCR [23].

### (e) Pine marten connectivity metrics

To compute pine marten connectivity, we use two results from the SCR analysis. First, the estimated spatial encounter model, which is a kernel that describes how space use declines with distance from the activity centre, and second, the realized density estimates for each pixel on the landscape. Following Sutherland *et al*. [24], we centre the kernel on each pixel and weight it by the realized density, and then compute the cumulative utilization of all pixels by all other pixels on the landscape. This produces a joint description of the population level relative frequency of pixel use across the landscape—or *density weighted connectivity*.

**Figure 1.**
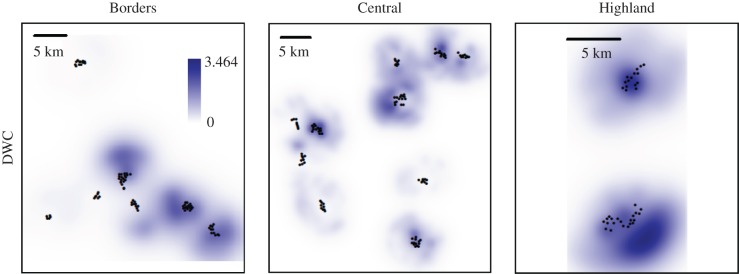
Pine marten density weighted connectivity surface for the Borders, Central and Highland study regions of Scotland with locations of multi-species detectors. (Online version in colour.)

The resulting derived measures are spatially explicit surfaces (100 m × 100 m) of pine marten density (DENS) and density weighted connectivity (DWC, figure 1). The former describes the distribution of activity centres, whereas the latter describes how intensely pine martens use parts of the landscape, and hence, is a candidate measure of exposure to pine marten impacts, whether mediated by consumptive or non-consumptive influences (predation hereafter). The interest was whether either of these measures explained variation in red or grey squirrel occurrence. Because each site was taken to represent an independent sampling unit, the average density and density weighted connectivity values within a 125 m buffer around each sampling site, i.e. larger than a typical squirrel home range radius of 100 m (as per [13]), were calculated.

### (f) Squirrel occupancy modelling

Like SCR, occupancy models are hierarchical models that jointly model the ecological process (occupancy, *ψ*) and the encounter process (detection probability, *p*) that accounts for imperfect detection through repeated visits. Using the detection–non detection squirrel data, we fit a series of single species occupancy models to investigate factors influencing squirrel occupancy, and in particular, to test hypotheses about the relationship between pine martens and red and grey squirrels. During the sampling period, we assumed negligible birth, recruitment, mortality and dispersal, i.e. closure was assumed. Thus, repeated visits are informative about detectability of the underlying occupancy state, detection probabilities can be estimated, and inference can be made about true occurrence and its determinants [25].

We modelled variation in detection as a function of two visit-specific covariates: VISIT, categorical covariate allowing detectability to vary by sampling occasion, and METHOD, a binary covariate indicating whether a camera and a feeder was deployed (1), or just a feeder (0). We also modelled between-region variation in detection using a site level categorical variable, REGION (noting that red squirrels occurred in all three regions, whereas grey squirrels are only present in CS and BO). We also modelled variation in detection as a function of forest attributes: per cent forest cover (COVER) and per cent forest cover that is broadleaved or mixed mainly broadleaved (BL), and pine marten metrics: pine marten density (DENS) and connectivity (DWC), all continuous site-level covariates summarized within a 100 m buffer of a site (electronic supplementary material, table S2).

We modelled variation in occupancy also as a function of per cent forest cover (COVER), per cent forest cover that is broadleaved or mixed mainly broadleaved (BL), and by region (REGION). Attempting to capture the competitive interaction of grey squirrels on reds, we included a measure of grey squirrel feeder use in the surrounding area (GS: the ratio of used/available feeders within a 500 m buffer of each feeder) in the red squirrel analysis. Finally, to test hypotheses about the effect of pine marten density and connectivity on squirrel occurrence, we also modelled occupancy as a function of the two covariates derived from the SCR analysis: DENS and DWC, respectively. DENS and DWC were never included in the same model.

We adopted a two-step approach to model selection. First, all combinations of the detection models, with no interactions, were fitted with two global models for the state process, i.e. a DENS and a DWC version of the most complex occupancy model. This resulted in 96 candidate detection models for each species (electronic supplementary material, file S3). Next, using the detection model(s) with most support, all biologically plausible combinations of occupancy models were fitted for each species. Models included no more than one two-way interaction resulting in 61 candidate occupancy models for grey squirrels and, with the addition of GS covariate, 132 candidate occupancy models for red squirrels (electronic supplementary material, file S4).

As with the SCR analysis, models were ranked and weighted according to AIC and in cases where no single model was overwhelmingly preferred, inference was based on model averaging the predictions of parameters of interest using AIC weights from all models. Models were fitted in R (R Core Team 2012) using the package UNMARKED [26]. We considered a model to be a competitor for drawing inference if parameters in the top model were not simply a subset of those in the competing models, as per Anderson & Burnham [27], resulting in one top detection model for each squirrel species.

## 3. Results

In total, there were 725 species detections at feeders (115 red squirrel, 101 grey squirrel and 509 pine marten) and 85 at trail cameras (26 red squirrel, 14 grey squirrel and 45 pine marten). Regional summaries of detections are provided in the electronic supplementary material, table S5. A total of 388 pine marten hair samples were successfully sex-typed and genotyped to individual level (76.5% of all samples), from which 42 unique genotypes were identified (19 female and 23 male). All loci were polymorphic with three to four alleles per locus, the probability of identity (PI_HW_) was less than 0.0001, and the sibling probability of identity (PI_sibs_) was 0.0143. On average, across the study period, individuals in BO, CS and HI were encountered 12.40, 11.00 and 7.09 times at an average of 6.80, 7.10 and 4.00 feeders, respectively, and 41 out of the 42 animals were encountered more than once.

### (a) Spatial capture–recapture

Pine marten density (ind km^−2^ of woodland), space use (km) and detection varied among the three regions. Density varied between region (cumulative AIC weight, or relative variable importance, of a region effect, *ω*^+^_D∼REGION_ = 0.82, table 1), and by sex (estimated probability of being a male, *ϕ*_male_ = 0.36 ± 0.13, table 1). Female marten density was about twice that of males (table 1). Densities were similar in the HI and CS regions and markedly lower in BO (table 1). Space use also differed both by region (*ω*^+^*_σ_*_∼REGION_ = 1.00) and sex (*ω*^+^*_σ_*_∼sex_ = 1.00), and was negatively correlated with density (table 1). Males moved more than twice as far as females, and spatial scale of movement was highest in the BO where density was lowest (*σ*_BO,female_ = 1.10 ± 0.11, *σ*_BO,male_ = 2.7 ± 0.24) compared to CS (*σ*_CS,female_ = 0.55 ± 0.06, *σ*_CS,male_ = 1.32 ± 0.12) and HI (*σ*_HI,female_ = 0.74 ± 0.12, *σ*_HI,male_ = 1.78 ± 0.35) regions. Finally, there was some support for variation in baseline detection probability by region (*ω*^+^_p∼REGION_ = 0.57) and by sex (*ω*^+^_p∼sex_ = 0.75, table 1). The probability of detecting a marten at its activity centre was highest in CS and lowest in HI (table 1).

**Table 1.** Model-averaged parameter estimates for male and female pine martens in the Borders, Central Scotland and the Highlands. Density is the estimated number of activity centres (individuals) per square km, detection (*p*) is the estimated probability of observing an individual at its activity centre, and sigma (*σ*) is the estimated spatial scale parameter that defines the distance over which the detection decreases.

		density (km^2^)		detection (*p*)		sigma (*σ*)	
sex	region	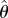	se( 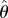 )	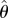	se( 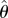 )	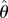	se( 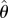 )
female	Borders	0.062	0.025	0.565	0.080	1.104	0.108
	Central	0.137	0.046	0.630	0.095	0.551	0.062
	Highlands	0.119	0.046	0.510	0.098	0.740	0.122
male	Borders	0.035	0.014	0.417	0.093	2.651	0.235
	Central	0.076	0.022	0.485	0.102	1.322	0.115
	Highlands	0.066	0.024	0.366	0.084	1.775	0.346

Based on the AIC ranked model set, we model-averaged predictions of density and density weighted connectivity surfaces, which were then used in the squirrel occupancy analyses. The range of estimated pixel-specific realized marten density (DENS), which was converted to individuals per square km, was 0.00–2.48 in BO, 0.00–5.86 in CS and 0.00–4.72 in HI. The range of estimated pixel-specific density weighted connectivity, which is a relative measure of utilization, was 0.06–2.08 in BO, 0.12–2.46 in CS and 1.17–3.06 in the HI.

### (b) Squirrel occupancy modelling

In the first step of our sequential modelling approach, a single preferred detection model was identified for each squirrel species in turn. For grey squirrels, the AIC-top detection model allowed for differences between regions (*ω*^+^*_p_*_∼REGION_ = 1.00) and visits (*ω*^+^*_p_*_∼VISIT_ = 1.00), and an effect of pine marten connectivity (*ω*^+^*_p_*_∼DWC_ = 0.83, *p*(REGION + DWC + VISIT), electronic supplementary material, table S6). For red squirrels, the AIC-top detection model also allowed for variation between regions (*ω*^+^*_p_*_∼REGION_ = 1.00) and visits (*ω*^+^*_p_*_∼VISIT_ = 1.00), and an effect of pine marten connectivity (*ω*^+^*_p_*_∼DWC_ = 1.00) but also allowed for differences in detectability depending on whether a camera was deployed or not (*ω*^+^*_p_*_∼METHOD_ = 1.00, *p*(REGION + DWC + METHOD + VISIT), electronic supplementary material, table S7). We note that although several models performed similarly, i.e. had less than two *Δ*AIC units, the AIC-top model was nested within all the models within four AIC units and thus contained uninformative parameters [28].

Grey squirrel detection probability was positively related to pine marten connectivity (*β*_DWC_ = 0.74 ± 0.26), was higher in CS than in BO (contrast relative to BO: *β*_CS_ = 0.74 ± 0.26), and increased through time by visit (contrasts of visit 2–4 relative to 1 from the top model: *β*_VISIT2_ = 1.36 ± 0.46, *β*_VISIT3_ = 1.708 ± 0.47, *β*_VISIT4_ = 3.185 ± 0.56; electronic supplementary material, table S6). For red squirrels, detection was negatively related to pine marten connectivity (*β*_DWC_ = −2.64 ± 0.42) (electronic supplementary material, table S8), was also higher in CS than in the BO (contrast relative to BO: *β*_CS_ = 6.22 ± 0.92), and also increased through time across visits (contrasts of visit 2–4 relative to 1 from the top model: *β*_VISIT2_ = 1.40 ± 0.54, *β*_VISIT3_ = 2.86 ± 0.58, *β*_VISIT4_ = 2.91 ± 0.58, *β*_VISIT5_ = 3.59 ± 0.75; electronic supplementary material, table S8). Red squirrel detection was also method-specific such that detection probability is much reduced when deploying feeders alone (*β*_HAIR_ = −6.59 ± 1.48; electronic supplementary material, table S7).

There was strong support for a negative relationship between grey squirrel occupancy and pine marten DWC, while no such relationship was evident for red squirrel occupancy (electronic supplementary material, tables S6 and S7). For grey squirrels, pine marten connectivity negatively affected grey squirrel occupancy: the slope of this relationship in BO was *β*_BO,DWC_ = −0.96 ± 0.37, and was stronger in CS (estimated difference in slopes from the top model, *β*_CS,DWC_ = −0.70 ± 0.63, electronic supplementary material, table S6). Occupancy also varied by region (*ω*^+^*_ψ_*_∼REGION_ = 1.00) and with the proportion of broadleaf cover (*ω*^+^*_ψ_*_∼BL_ = 0.83), and there was substantial support for an interaction, i.e. that the slopes of the broadleaf relationship were different between the regions (*ω*^+^*_ψ_*_∼REGION:BL_ = 0.76, electronic supplementary material, table S6). All else equal, grey squirrel occupancy was lower in CS than in BO (from the top model with BO as the reference level: *β*_CS_ = −1.282 ± 0.7) and was positively related to BL (top model: *β*_BL_ = 0.56 ± 0.57, electronic supplementary material, table S6).

**Figure 2.**
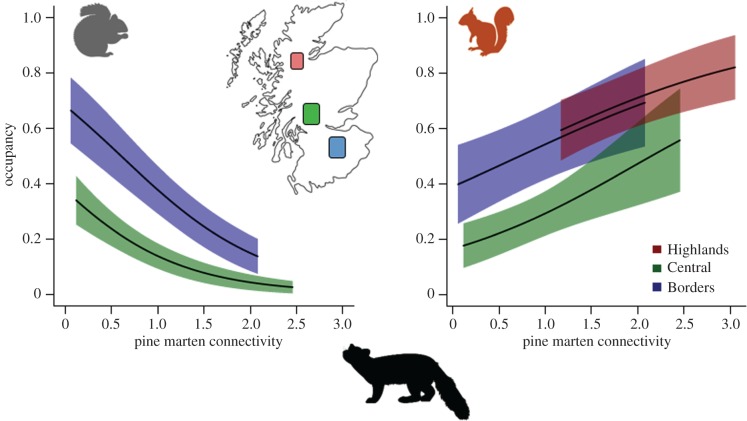
Model-averaged predictions of the relationships between occupancy and pine marten density weighted connectivity for grey squirrels and red squirrels in the Borders, Central and Highland regions of Scotland.

For red squirrels, the opposite effect of pine marten connectivity was observed, i.e. DWC positively affected red squirrel occupancy: the common slope of this relationship is *β*_DWC_ = 0.81 ± 0.32 (electronic supplementary material, table S7). Occupancy was also positively related to the proportion of total cover (*ω*^+^*_ψ_*_∼COVER_ = 0.91, top model: *β*_COVER_ = 3.21 ± 1.46) and negatively associated with the per cent of broadleaf cover (*ω*^+^*_ψ_*_∼BL_ = 0.85, top model: *β*_BL_ = −1.18 ± 0.47, electronic supplementary material, table S7). Model-averaged predictions of the occupancy connectivity relationships on the probability scale for both species are shown in figure 2 using partial regression holding all other covariates at region-specific average (median) conditions.

## 4. Discussion

We found unequivocal support for the hypothesis that a recovering native predator density is modifying competition between two closely related native and non-native prey species. As with the virus that links the two species, the impact of exposure to a native predator was highly asymmetrical, but in the opposite direction to that of disease. Non-native grey squirrel presence was strongly negatively affected by connectivity to individual pine martens, whereas the presence of the native red squirrel was seemingly positively affected.

### (a) Study design

Our evidence is correlative rather than experimental but derived from a structured survey considering key covariates, and the sampling methods used account for variation in detectability of the focal species. Central to the design was the stratified sampling in three regions, each with a contrasting time since recolonization by pine martens, and thus differing lengths of time for which squirrel populations have been exposed to pine marten predation. It is important to note that our inferences are not solely based on between-region differences in squirrel occupancy, as our analysis included a region-specific intercept to account for this, but rather on the large between- and intra-region variation in pine marten density reflected in marten connectivity and its relationship with squirrel occupancy.

Our study meets the high standard of statistical rigour required of studies seeking to inform policy on controversial wildlife management issues. Through embracing probabilistic methods to account for variability in detection probability in field data, we elevated the robustness of evidence relative to previous studies using naive occupancy and indices of abundance [9]. For instance, had we not used trail cameras in addition to feeders, red squirrel probability of detection would have been artificially high which, if unaccounted for, would have led to low naive occupancy estimates and thus a spurious negative effect of pine martens on red squirrels.

### (b) Marten density versus connectivity to martens

While there is no expectation that the abundance of pine martens, a generalist predator, should be strongly affected by the abundance of any single prey species, variation in predator abundance is likely to regulate the abundance of prey populations [29]. In describing correlative patterns in detection rates of pine marten, and red (positive) and grey (negative) squirrels, Sheehy & Lawton [9] concluded that inference based on detection rates was overly simplistic and that marten abundance likely influences the interactions between the three species. Here we directly address this issue using recently developed spatial capture–recapture methods to generate spatially explicit estimates of marten density and density weighted connectivity [24]. The important distinction here is that density surfaces describe the spatial distribution of activity centres, ignoring available information about how individuals use space. By contrast, density weighted connectivity incorporates information about marten space use and describes how connected any location on the landscape is to the population of marten in the area, i.e. for squirrels, it is a measure of overall exposure to predation. Although only recently proposed [24], density weighted connectivity provides a more intuitive measure for how frequently species interact than a description of where activity centres are located.

In direct support of the hypothesis that pine martens have the potential to naturally control populations of invasive grey squirrels [9], our analyses demonstrate that exposure to pine marten (i.e. connectivity) clearly suppresses grey squirrel populations in Scotland. Moreover, we provide equally compelling evidence that this negative effect is not realized in red squirrels. In fact, we found a positive effect of pine marten connectivity on red squirrels in all three regions. This positive effect is likely a consequence of the suppression of grey squirrel populations by pine martens as demonstrated in BO and CS, and is suggestive of predator modified competition. Although the lack of any interactions between region and pine marten, DWC suggests the positive relationship extends to the HI region, where grey squirrels are absent, this likely reflects a lack of statistical power in HI (36 detectors, compared to 80 and 107 in BO and CS, respectively), thus the common relationship is dominated by BO and CS.

### (c) Hint at proximal cause for differential impact

Our findings that grey and red squirrel detectability increased and decreased with pine marten DWC (*β*_DWC_ = 0.74 ± 0.26 and *β*_DWC_ = −2.64 ± 0.42, respectively) provide some indication as to the proximal basis for the asymmetrical impact of pine martens on red and grey squirrels. The detection component of our occupancy models imply that a considerable behavioural difference exists between red and grey squirrels, such that red squirrels, despite being present, were less likely to visit feeders in sites with high pine marten connectivity, i.e. they respond in accordance with exposure to predation risk. Conversely, grey squirrels, given they were present, were more likely to use feeders in sites with high pine marten connectivity, suggesting a maladaptive response to a predator with which they share no coevolutionary history. Sheehy *et al.* [30] reported that grey squirrels occurred in 15.6% of pine marten scats in Ireland compared to 2.5% occurrence of red squirrels. This difference in the frequency of occurrence between red and grey squirrels may also reflect a higher vulnerability of grey squirrels to direct predation by pine martens as a result of predator naivety; however estimates of predation rates in areas where grey squirrels are available as prey items are lacking.

### (d) Nature of predation impact: extirpation versus fugitive coexistence

Occupancy models predict near zero occupancy probabilities of grey squirrel presence in those portions of landscapes with the highest pine marten connectivity and severely depressed, but above zero, occupancy probabilities elsewhere. Whether the recovery of the pine marten will lead to the eventual extirpation of grey squirrels, and of the SQPV that it transmits to red squirrels, or to a situation of coexistence with grey squirrels persisting fugitively at low density in parts of the landscape not used by pine martens cannot be determined from the snapshot data gathered here.

In our survey, areas with the highest pine marten connectivity values were encountered in the CS region where martens have been present for 8–14 years. However, even in this region where the relatively slow growing marten population had time to increase, there remained many portions of the landscape where marten density is presently too low for region-wide grey squirrel extinction to occur. However, while grey squirrel occupancy was positively related to broadleaf forest cover in the BO region most recently colonized by pine martens, this was not the case in CS. A tentative interpretation of the interaction between REGION and broadleaf forest cover in models predicting grey squirrel occupancy influence is that, over time, pine martens suppress grey squirrel numbers even in their preferred habitats.

### (e) Implications for disease-mediated apparent competition

Despite the uncertainty on whether the return of the pine marten will ultimately lead to the extirpation or suppression of grey squirrel abundance, it will likely profoundly alter the overall competitive interaction between the squirrel species through its impact on SQPV dynamics. General understanding of directly transmitted diseases, specific models of SQPV spatial dynamics [14] and field evidence that grey squirrel control can lead to a reduction in disease prevalence in residual grey populations [31] all suggest that pine-marten-induced reductions in grey squirrel density will affect SQPV transmission. Depending on the size of grey squirrel refuges from pine marten predation in, for example, urban areas, their connectivity through habitat corridors and chance events, pine marten predation on grey squirrels might either reduce or even eradicate the pathogen in local grey squirrel populations. Establishing whether this expected reduction is sufficient to preclude the transmission of SQPV to immunologically susceptible red squirrel populations [32] is an urgent applied research priority. Central to current red squirrel management strategies is the creation of refuges from grey squirrel-mediated infection with control buffers where intensive grey squirrel culling is instigated. Siting such refuges in areas where pine martens have fully recovered might be one means to make such long-term management feasible.

### (f) Management implications

Our evidence that, in addition to their intrinsic value, pine martens provide an ecosystem service by suppressing invasive grey squirrel populations, which has important management implications. Substantial funds are spent annually in the UK to control grey squirrels, for the benefit of red squirrel conservation, and particularly in the southern half of Britain, to limit the damage to growing deciduous timber caused by grey squirrels [33]. The pine marten is already heavily suppressing grey squirrel populations where they are well established, and presumably this influence will spread spatially over time.

The pine marten's range in the UK is currently expanding southwards through Scotland and into the north of England but this is a slow, gradual process, commensurate with the low fecundity of this species. This range expansion will take place in areas where conflicts surrounding predators can be locally acute and associated with the illegal persecution of protected predator species [34]. It is as yet unknown whether the pine marten, as a recovering predator, is a victim of such persecution and what contribution this makes to slowing down the spread of the species.

There have been several reintroductions of pine martens in Britain, including translocations in the BO region of our survey, and official reintroductions in southwest Scotland, and a population reinforcement in Wales. Our analyses suggest that the impact of these reintroductions on grey squirrels will only become substantial when pine marten density has risen. Such densities are to be expected in established populations and behind the frontier of naturally expanding ranges.

Most of the residual range of the red squirrel in northern Britain has been recolonized by the pine marten, and our findings provide confidence that the return of the pine marten will not have a detrimental impact on red squirrels, even where pine martens reach average natural densities of 0.19 ind km^−2^ of woodland as seen in the Highlands of Scotland. Indeed, a detrimental impact would not be the expected outcome of interactions between two species that share a coevolutionary history. By contrast, it is not unlikely that other prey species may be affected by the return of a long absent native predator. Evidence from Fennoscandia suggests that the pine marten is likely to affect the reproductive success of some ground nesting bird species in years of low vole abundance, with transient impacts on abundance (e.g. [35]). Future investigations as to the impact of pine martens on the capercaillie, *Tetrao urogallus*, a species endangered in Scotland's fragmented forest as a result of climate change [36] and increasing predation by generalists, ought to adopt the rigorous density estimates we strove for here rather than using uncalibrated indices (e.g. [37]).

## 5. Conclusion

Hitherto, both resource and disease-mediated apparent competition between red and grey squirrels have been well documented. Our results demonstrate how the addition of a second, indirect, interaction involving a recovering predator reverses the expected outcome of resource and disease-mediated competition. Where the native predator recovery is more advanced, the native squirrel species now occupies a greater portion of the landscape than non-native grey squirrels which are predicted to only persist in landscape refugia where the predators are scarce. As such we present a rare demonstration of predator-mediated competition in terrestrial mammals also linked by exploitative and disease-mediated apparent competition.

## Supplementary Material

Table S1 from The enemy of my enemy is my friend: Native pine marten recovery reverses the decline of the red squirrel by suppressing grey squirrel populations

## Supplementary Material

Table S2 from The enemy of my enemy is my friend: Native pine marten recovery reverses the decline of the red squirrel by suppressing grey squirrel populations

## Supplementary Material

File S3 from The enemy of my enemy is my friend: Native pine marten recovery reverses the decline of the red squirrel by suppressing grey squirrel populations

## Supplementary Material

File S4 from The enemy of my enemy is my friend: Native pine marten recovery reverses the decline of the red squirrel by suppressing grey squirrel populations

## Supplementary Material

Table S5 from The enemy of my enemy is my friend: Native pine marten recovery reverses the decline of the red squirrel by suppressing grey squirrel populations

## Supplementary Material

Table S6 from The enemy of my enemy is my friend: Native pine marten recovery reverses the decline of the red squirrel by suppressing grey squirrel populations

## Supplementary Material

Table S7 from The enemy of my enemy is my friend: Native pine marten recovery reverses the decline of the red squirrel by suppressing grey squirrel populations

## Supplementary Material

File S8 from The enemy of my enemy is my friend: Native pine marten recovery reverses the decline of the red squirrel by suppressing grey squirrel populations

## Supplementary Material

Data S9 from The enemy of my enemy is my friend: Native pine marten recovery reverses the decline of the red squirrel by suppressing grey squirrel populations

## Supplementary Material

Data S10 from The enemy of my enemy is my friend: Native pine marten recovery reverses the decline of the red squirrel by suppressing grey squirrel populations

## Supplementary Material

Data S11 from The enemy of my enemy is my friend: Native pine marten recovery reverses the decline of the red squirrel by suppressing grey squirrel populations

## Supplementary Material

Data S12 from The enemy of my enemy is my friend: Native pine marten recovery reverses the decline of the red squirrel by suppressing grey squirrel populations

## Data accessibility

The datasets supporting this article have been uploaded to Dryad: http://dx.doi.org/10.5061/dryad.478kp63 [38].
