# Supplementary material for: The enemy of my enemy is my friend: native pine marten recovery reverses the decline of the red squirrel by suppressing grey squirrel populations
Source: Proc Biol Sci. 2018 Mar 7;285(1874):20172603. doi: 10.1098/rspb.2017.2603 (PMC5879625; doi:10.1098/rspb.2017.2603)
Supplement: Table S1 from The enemy of my enemy is my friend: Native pine marten recovery reverses the decline of the red squirrel by suppressing grey squirrel populations [file rspb20172603supp1.pdf]

region	grid cell	woodland (ha)	broadleaf %	sampled area (ha)	broadleaf %
CS	1B	221	0.46	61	0.21
	1C	230	0.43	99	0.48
	1D	102	0.65	56	0.50
	1E	177	0.91	110	0.99
	1F	212	0.76	171	0.78
	1G	176	0.73	131	0.64
	1H	140	0.70	76	0.69
	1J	118	0.76	89	0.78
	1K	113	0.90	54	0.93
	1Q	87	1.00	53	1.00
BO	2L	160	0.15	83	0.06
	2M	247	0.13	152	0.09
	2N	129	0.06	90	0.01
	2P	68	0.37	45	0.02
	2X	170	0.45	30	0.68
	2Y	140	0.49	69	0.43
	2Z	293	0.00	188	0.00
HI	CY	222	0.16	155	0.10
	DD	447	0.13	297	0.09

Table S1. The amount of woodland in hectres (ha), the percentage of woodland composed of broadleaf habitat , the sampled area, and the percentage of sampled area composed of broadleaf habitat in each grid cell selected for sampling in the Central (CS), Borders (BO) and Highlands (HI) regions of Scotland.
