# Supplementary material for: The enemy of my enemy is my friend: native pine marten recovery reverses the decline of the red squirrel by suppressing grey squirrel populations
Source: Proc Biol Sci. 2018 Mar 7;285(1874):20172603. doi: 10.1098/rspb.2017.2603 (PMC5879625; doi:10.1098/rspb.2017.2603)
Supplement: Table S2 from The enemy of my enemy is my friend: Native pine marten recovery reverses the decline of the red squirrel by suppressing grey squirrel populations [file rspb20172603supp2.pdf]

Table S2. Mean and range values for site covariates used to model red and grey squirrel probabilities of detection and occupancy in the Borders, Central and Highland regions. DWC = pine marten density weighted connectivity, DENS = pine marten density, BL = portion of habitat which was broadleaved, COVER = portion of site which contained trees, GS = proportion of feeders used by grey squirrels/all feeders within 500m

		DWC		DENS		BL		COVER		GS	
REGION	n	x̄	range	x̄	range	x̄	range	x̄	range	x̄	range
BO	80	1.04	0.06 – 2.08	0.08	0.00 – 2.48	0.15	0.00 – 1.00	0.92	0.32 – 1.00	0.39	0.00 – 1.00
CS	107	1.02	0.12 – 2.46	0.18	0.00 – 5.86	0.69	0.00 – 1.00	0.90	0.42 – 1.00	0.18	0.00 – 1.00
HI	36	2.19	1.17 – 3.06	0.56	0.00 – 4.72	0.06	0.00 – 1.00	0.89	0.00 – 1.00	-	-
