# Supplementary material for: The enemy of my enemy is my friend: native pine marten recovery reverses the decline of the red squirrel by suppressing grey squirrel populations
Source: Proc Biol Sci. 2018 Mar 7;285(1874):20172603. doi: 10.1098/rspb.2017.2603 (PMC5879625; doi:10.1098/rspb.2017.2603)
Supplement: File S3 from The enemy of my enemy is my friend: Native pine marten recovery reverses the decline of the red squirrel by suppressing grey squirrel populations [file rspb20172603supp3.pdf]

```
library(unmarked)
library(raster)


setwd()
load("aveDensity.Rdata")  # output file from scr, i.e. need to have run scr analysis first
load("aveDWC.Rdata")      # also scr output file


detections <-   read.csv("detections_grey.csv")
grey <- subset(detections, Species %in% "grey")
tmp1 <- cbind(extract(aveDensity[[1]],as.matrix(grey[,c("X","Y")]/1000)),
        extract(aveDensity[[3]],as.matrix(grey[,c("X","Y")]/1000)))
tmp2 <- cbind(extract(aveDWC[[1]],    as.matrix(grey[,c("X","Y")]/1000)),
        extract(aveDWC[[3]],    as.matrix(grey[,c("X","Y")]/1000)))


grey$dens <- scale(log(apply(tmp1,1,sum,na.rm=T) * 1/(0.1^2)))
grey$dwc <- scale(apply(tmp2,1,sum,na.rm=T)             )
grey$dens <- apply(tmp1,1,sum,na.rm=T) * 1/(0.1^2)
grey$dwc <- apply(tmp2,1,sum,na.rm=T)


rmv <- which(apply(is.na(grey[,paste0("visit",1:4)]),1,sum)%in%4)


obsCovs <- list()
obsCovs[["method"]] <- grey[-rmv,paste0("tCov",1:4)]
obsCovs[["visit"]]  <- matrix(factor(col(obsCovs[[1]])),
                  nrow(obsCovs[[1]]),ncol(obsCovs[[1]]))
colnames(obsCovs[["method"]]) <- paste0("method.",1:4)
colnames(obsCovs[["visit"]])  <- paste0("visit.", 1:4)


grey.umf <- unmarkedFrameOccu(y=grey[-rmv,paste0("visit",1:4)],
                  siteCovs = grey[-rmv,c("region","dens", "dwc", "cover", "bl")],
                  obsCovs = obsCovs)



#####################################################################################
## Stage one: compare detection models using a flexible occupancy structure
grey.p.mods <- list(#~p
```

#psi~.+density

#single terms

"p(1) psi(region+cover+bl + dens)" = occu(~1 ~region+cover+bl+dens, grey.umf),

"p(region) psi(region+cover+bl + dens)" = occu(~region ~region+cover+bl+dens, grey.umf),

"p(method) psi(region+cover+bl + dens)" = occu(~method ~region+cover+bl+dens, grey.umf),

"p(cover) psi(region+cover+bl + dens)" = occu(~cover ~region+cover+bl+dens, grey.umf),

"p(bl) psi(region+cover+bl + dens)" = occu(~bl ~region+cover+bl+dens, grey.umf),

"p(dens) psi(region+cover+bl + dens)" = occu(~dens ~region+cover+bl+dens, grey.umf),

"p(dwc) psi(region+cover+bl + dens)" = occu(~dwc ~region+cover+bl+dens, grey.umf),

"p(visit) psi(region+cover+bl + dens)" = occu(~visit ~region+cover+bl+dens, grey.umf),

#two terms

"p(region+method) psi(region+cover+bl + dens)" = occu(~region+method ~region+cover+bl+dens, grey.umf),

"p(region+cover) psi(region+cover+bl + dens)" = occu(~region+cover ~region+cover+bl+dens, grey.umf),

"p(region+bl) psi(region+cover+bl + dens)" = occu(~region+bl ~region+cover+bl+dens, grey.umf),

"p(region+dens) psi(region+cover+bl + dens)" = occu(~region+dens ~region+cover+bl+dens, grey.umf),

"p(region+dwc) psi(region+cover+bl + dens)" = occu(~region+dwc ~region+cover+bl+dens, grey.umf),

"p(region+visit) psi(region+cover+bl + dens)" = occu(~region+visit ~region+cover+bl+dens, grey.umf),

"p(method+cover) psi(region+cover+bl + dens)" = occu(~method+cover ~region+cover+bl+dens, grey.umf),

"p(method+bl) psi(region+cover+bl + dens)" = occu(~method+bl ~region+cover+bl+dens, grey.umf),

"p(method+dens) psi(region+cover+bl + dens)" = occu(~method+dens ~region+cover+bl+dens, grey.umf),

"p(method+dwc) psi(region+cover+bl + dens)" = occu(~method+dwc ~region+cover+bl+dens, grey.umf),

"p(method+visit) psi(region+cover+bl + dens)" = occu(~method+visit ~region+cover+bl+dens, grey.umf),

"p(cover+bl) psi(region+cover+bl + dens)" = occu(~cover+bl ~region+cover+bl+dens, grey.umf),

"p(cover+dens) psi(region+cover+bl + dens)" = occu(~cover+dens ~region+cover+bl+dens, grey.umf),

"p(cover+dwc) psi(region+cover+bl + dens)" = occu(~cover+dwc ~region+cover+bl+dens, grey.umf),

"p(cover+visit) psi(region+cover+bl + dens)" = occu(~cover+visit ~region+cover+bl+dens, grey.umf),

"p(bl+dens) psi(region+cover+bl + dens)" = occu(~bl+dens ~region+cover+bl+dens, grey.umf),

"p(bl+dwc) psi(region+cover+bl + dens)" = occu(~bl+dwc ~region+cover+bl+dens, grey.umf),

"p(bl+visit) psi(region+cover+bl + dens)" = occu(~bl+visit ~region+cover+bl+dens, grey.umf),

"p(dens+visit) psi(region+cover+bl + dens)" = occu(~dens+visit ~region+cover+bl+dens, grey.umf),

"p(dwc+visit) psi(region+cover+bl + dens)" = occu(~dwc+visit ~region+cover+bl+dens, grey.umf),

#three terms

"p(region+method+cover) psi(region+cover+bl + dens)" = occu(~region+method+cover ~region+cover+bl+dens, grey.umf),

"p(region+method+bl) psi(region+cover+bl + dens)" = occu(~region+method+bl ~region+cover+bl+dens, grey.umf),

"p(region+method+dens) psi(region+cover+bl + dens)" = occu(~region+method+dens ~region+cover+bl+dens, grey.umf),

"p(region+method+dwc) psi(region+cover+bl + dens)" = occu(~region+method+dwc ~region+cover+bl+dens, grey.umf),

"p(region+method+visit) psi(region+cover+bl + dens)" = occu(~region+method+visit ~region+cover+bl+dens, grey.umf),

"p(region+cover+bl) psi(region+cover+bl + dens)" = occu(~region+cover+bl ~region+cover+bl+dens, grey.umf),

"p(region+cover+dens) psi(region+cover+bl + dens)" = occu(~region+cover+dens ~region+cover+bl+dens, grey.umf),

"p(region+cover+dwc) psi(region+cover+bl + dens)" = occu(~region+cover+dwc ~region+cover+bl+dens, grey.umf),

"p(region+cover+visit) psi(region+cover+bl + dens)" = occu(~region+cover+visit ~region+cover+bl+dens, grey.umf),

"p(region+bl+dens) psi(region+cover+bl + dens)" = occu(~region+bl+dens ~region+cover+bl+dens, grey.umf),

"p(region+bl+dwc) psi(region+cover+bl + dens)" = occu(~region+bl+dwc ~region+cover+bl+dens, grey.umf),

"p(region+bl+visit) psi(region+cover+bl + dens)" = occu(~region+bl+visit ~region+cover+bl+dens, grey.umf),

"p(region+dens+visit) psi(region+cover+bl + dens)" = occu(~region+dens+visit ~region+cover+bl+dens, grey.umf),

"p(region+dwc+visit) psi(region+cover+bl + dens)" = occu(~region+dwc+visit ~region+cover+bl+dens, grey.umf),

"p(method+cover+bl) psi(region+cover+bl + dens)" = occu(~method+cover+bl ~region+cover+bl+dens, grey.umf),

"p(method+cover+dens) psi(region+cover+bl + dens)" = occu(~region+cover+dens ~region+cover+bl+dens, grey.umf),

"p(method+cover+dwc) psi(region+cover+bl + dens)" = occu(~method+cover+dwc ~region+cover+bl+dens, grey.umf),

"p(method+cover+visit) psi(region+cover+bl + dens)" = occu(~method+cover+visit ~region+cover+bl+dens, grey.umf),

"p(method+bl+dens) psi(region+cover+bl + dens)" = occu(~method+bl+dens ~region+cover+bl+dens, grey.umf),

"p(method+bl+dwc) psi(region+cover+bl + dens)" = occu(~method+bl+dwc ~region+cover+bl+dens, grey.umf),

"p(method+bl+visit) psi(region+cover+bl + dens)" = occu(~method+bl+visit ~region+cover+bl+dens, grey.umf),

"p(method+dens+visit) psi(region+cover+bl + dens)" = occu(~method+dens+visit ~region+cover+bl+dens, grey.umf),

"p(method+dwc+visit) psi(region+cover+bl + dens)" = occu(~method+dwc+visit ~region+cover+bl+dens, grey.umf),

"p(cover+bl+dens) psi(region+cover+bl + dens)" = occu(~cover+bl+dens ~region+cover+bl+dens, grey.umf),

"p(cover+bl+dwc) psi(region+cover+bl + dens)" = occu(~cover+bl+dwc ~region+cover+bl+dens, grey.umf),

"p(cover+bl+visit) psi(region+cover+bl + dens)" = occu(~cover+bl+visit ~region+cover+bl+dens, grey.umf),

"p(cover+dens+visit) psi(region+cover+bl + dens)" = occu(~cover+dens+visit ~region+cover+bl+dens, grey.umf),

"p(cover+dwc+visit) psi(region+cover+bl + dens)" = occu(~cover+dwc+visit ~region+cover+bl+dens, grey.umf),

"p(bl+dens+visit) psi(region+cover+bl + dens)" = occu(~bl+dens+visit ~region+cover+bl+dens, grey.umf),

"p(bl+dwc+visit)   psi(region+cover+bl + dens)" = occu(~bl+dwc+visit   ~region+cover+bl+dens, grey.umf),

#four terms

"p(region+method+cover+bl)   psi(region+cover+bl + dens)" = occu(~region+method+cover+bl
~region+cover+bl+dens, grey.umf),

"p(region+method+cover+dens)   psi(region+cover+bl + dens)" = occu(~region+method+cover+dens
~region+cover+bl+dens, grey.umf),

"p(region+method+cover+dwc)   psi(region+cover+bl + dens)" = occu(~region+method+cover+dwc
~region+cover+bl+dens, grey.umf),

"p(region+method+cover+visit)   psi(region+cover+bl + dens)" = occu(~region+method+cover+visit
~region+cover+bl+dens, grey.umf),

"p(region+method+bl+dens)   psi(region+cover+bl + dens)" = occu(~region+method+bl+dens
~region+cover+bl+dens, grey.umf),

"p(region+method+bl+dwc)   psi(region+cover+bl + dens)" = occu(~region+method+bl+dwc
~region+cover+bl+dens, grey.umf),

"p(region+method+bl+visit)   psi(region+cover+bl + dens)" = occu(~region+method+bl+visit   ~region+cover+bl+dens,
grey.umf),

"p(region+method+dens+visit)   psi(region+cover+bl + dens)" = occu(~region+method+dens+visit
~region+cover+bl+dens, grey.umf),

"p(region+method+dwc+visit)   psi(region+cover+bl + dens)" = occu(~region+method+dwc+visit
~region+cover+bl+dens, grey.umf),

"p(region+cover+bl+dens)   psi(region+cover+bl + dens)" = occu(~region+cover+bl+dens   ~region+cover+bl+dens,
grey.umf),

"p(region+cover+bl+dwc)   psi(region+cover+bl + dens)" = occu(~region+cover+bl+dwc   ~region+cover+bl+dens,
grey.umf),

"p(region+cover+bl+visit)   psi(region+cover+bl + dens)" = occu(~region+cover+bl+visit   ~region+cover+bl+dens,
grey.umf),

"p(region+cover+dens+visit)   psi(region+cover+bl + dens)" = occu(~region+cover+dens+visit   ~region+cover+bl+dens,
grey.umf),

"p(region+cover+dwc+visit)   psi(region+cover+bl + dens)" = occu(~region+cover+dwc+visit   ~region+cover+bl+dens,
grey.umf),

"p(region+bl+dens+visit)   psi(region+cover+bl + dens)" = occu(~region+bl+dens+visit   ~region+cover+bl+dens,
grey.umf),

"p(region+bl+dwc+visit)   psi(region+cover+bl + dens)" = occu(~region+bl+dwc+visit   ~region+cover+bl+dens,
grey.umf),

"p(method+cover+bl+dens)   psi(region+cover+bl + dens)" = occu(~method+cover+bl+dens
~region+cover+bl+dens, grey.umf),

"p(method+cover+bl+dwc)   psi(region+cover+bl + dens)" = occu(~method+cover+bl+dwc
~region+cover+bl+dens, grey.umf),

"p(method+cover+bl+visit)   psi(region+cover+bl + dens)" = occu(~method+cover+bl+visit   ~region+cover+bl+dens,
grey.umf),

"p(method+cover+dens+visit)   psi(region+cover+bl + dens)" = occu(~method+cover+dens+visit
~region+cover+bl+dens, grey.umf),

"p(method+cover+dwc+visit)   psi(region+cover+bl + dens)" = occu(~method+cover+dwc+visit
~region+cover+bl+dens, grey.umf),

"p(method+bl+dens+visit)   psi(region+cover+bl + dens)" = occu(~method+bl+dens+visit   ~region+cover+bl+dens, grey.umf),

"p(method+bl+dwc+visit)   psi(region+cover+bl + dens)" = occu(~method+bl+dwc+visit   ~region+cover+bl+dens, grey.umf),

"p(cover+bl+dens+visit)   psi(region+cover+bl + dens)" = occu(~cover+bl+dens+visit   ~region+cover+bl+dens, grey.umf),

"p(cover+bl+dwc+visit)   psi(region+cover+bl + dens)" = occu(~cover+bl+dwc+visit   ~region+cover+bl+dens, grey.umf),

#five terms

"p(region+method+cover+bl+dens)   psi(region+cover+bl + dens)" = occu(~region+method+cover+bl+dens ~region+cover+bl+dens, grey.umf),

"p(region+method+cover+bl+dwc)   psi(region+cover+bl + dens)" = occu(~region+method+cover+bl+dwc ~region+cover+bl+dens, grey.umf),

"p(region+method+cover+bl+visit)   psi(region+cover+bl + dens)" = occu(~region+method+cover+bl+visit ~region+cover+bl+dens, grey.umf),

"p(region+method+cover+dens+visit)   psi(region+cover+bl + dens)" = occu(~region+method+cover+dens+visit ~region+cover+bl+dens, grey.umf),

"p(region+method+cover+dwc+visit)   psi(region+cover+bl + dens)" = occu(~region+method+cover+dwc+visit ~region+cover+bl+dens, grey.umf),

"p(region+method+bl+dens+visit)   psi(region+cover+bl + dens)" = occu(~region+method+bl+dens+visit ~region+cover+bl+dens, grey.umf),

"p(region+method+bl+dwc+visit)   psi(region+cover+bl + dens)" = occu(~region+method+bl+dwc+visit ~region+cover+bl+dens, grey.umf),

"p(region+cover+bl+dens+visit)   psi(region+cover+bl + dens)" = occu(~region+cover+bl+dens+visit ~region+cover+bl+dens, grey.umf),

"p(region+cover+bl+dwc+visit)   psi(region+cover+bl + dens)" = occu(~region+cover+bl+dwc+visit ~region+cover+bl+dens, grey.umf),

"p(method+cover+bl+dens+visit)   psi(region+cover+bl + dens)" = occu(~method+cover+bl+dens+visit ~region+cover+bl+dens, grey.umf),

"p(method+cover+bl+dwc+visit)   psi(region+cover+bl + dens)" = occu(~method+cover+bl+dwc+visit ~region+cover+bl+dens, grey.umf),

#six terms

"p(region+method+cover+bl+dens+visit)psi(region+cover+bl + dens)" = occu(~region+method+cover+bl+dens+visit~region+cover+bl+dens, grey.umf),

"p(region+method+cover+bl+dwc+visit) psi(region+cover+bl + dens)" = occu(~region+method+cover+bl+dwc+visit ~region+cover+bl+dens, grey.umf),

#psi~.+dwc

#single terms

"p(1)   psi(region+cover+bl + dwc)" = occu(~1   ~region+cover+bl+dwc, grey.umf),

"p(region)   psi(region+cover+bl + dwc)" = occu(~region ~region+cover+bl+dwc, grey.umf),

"p(method)   psi(region+cover+bl + dwc)" = occu(~method ~region+cover+bl+dwc, grey.umf),

```
"p(cover)            psi(region+cover+bl + dwc)" = occu(~cover   ~region+cover+bl+dwc, grey.umf),
"p(bl)               psi(region+cover+bl + dwc)" = occu(~bl      ~region+cover+bl+dwc, grey.umf),
"p(dens)             psi(region+cover+bl + dwc)" = occu(~dens    ~region+cover+bl+dwc, grey.umf),
"p(dwc)              psi(region+cover+bl + dwc)" = occu(~dwc     ~region+cover+bl+dwc, grey.umf),
"p(visit)            psi(region+cover+bl + dwc)" = occu(~visit   ~region+cover+bl+dwc, grey.umf),

#two terms
"p(region+method)    psi(region+cover+bl + dwc)" = occu(~region+method  ~region+cover+bl+dwc, grey.umf),
"p(region+cover)     psi(region+cover+bl + dwc)" = occu(~region+cover   ~region+cover+bl+dwc, grey.umf),
"p(region+bl)        psi(region+cover+bl + dwc)" = occu(~region+bl      ~region+cover+bl+dwc, grey.umf),
"p(region+dens)      psi(region+cover+bl + dwc)" = occu(~region+dens    ~region+cover+bl+dwc, grey.umf),
"p(region+dwc)       psi(region+cover+bl + dwc)" = occu(~region+dwc     ~region+cover+bl+dwc, grey.umf),
"p(region+visit)     psi(region+cover+bl + dwc)" = occu(~region+visit   ~region+cover+bl+dwc, grey.umf),
"p(method+cover)     psi(region+cover+bl + dwc)" = occu(~method+cover   ~region+cover+bl+dwc, grey.umf),
"p(method+bl)        psi(region+cover+bl + dwc)" = occu(~method+bl      ~region+cover+bl+dwc, grey.umf),
"p(method+dens)      psi(region+cover+bl + dwc)" = occu(~method+dens    ~region+cover+bl+dwc, grey.umf),
"p(method+dwc)       psi(region+cover+bl + dwc)" = occu(~method+dwc     ~region+cover+bl+dwc, grey.umf),
"p(method+visit)     psi(region+cover+bl + dwc)" = occu(~method+visit   ~region+cover+bl+dwc, grey.umf),
"p(cover+bl)         psi(region+cover+bl + dwc)" = occu(~cover+bl       ~region+cover+bl+dwc, grey.umf),
"p(cover+dens)       psi(region+cover+bl + dwc)" = occu(~cover+dens     ~region+cover+bl+dwc, grey.umf),
"p(cover+dwc)        psi(region+cover+bl + dwc)" = occu(~cover+dwc      ~region+cover+bl+dwc, grey.umf),
"p(cover+visit)      psi(region+cover+bl + dwc)" = occu(~cover+visit    ~region+cover+bl+dwc, grey.umf),
"p(bl+dens)          psi(region+cover+bl + dwc)" = occu(~bl+dens        ~region+cover+bl+dwc, grey.umf),
"p(bl+dwc)           psi(region+cover+bl + dwc)" = occu(~bl+dwc         ~region+cover+bl+dwc, grey.umf),
"p(bl+visit)         psi(region+cover+bl + dwc)" = occu(~bl+visit       ~region+cover+bl+dwc, grey.umf),
"p(dens+visit)       psi(region+cover+bl + dwc)" = occu(~dens+visit     ~region+cover+bl+dwc, grey.umf),
"p(dwc+visit)        psi(region+cover+bl + dwc)" = occu(~dwc+visit      ~region+cover+bl+dwc, grey.umf),

#three terms
"p(region+method+cover)    psi(region+cover+bl + dwc)" = occu(~region+method+cover  ~region+cover+bl+dwc,
grey.umf),
"p(region+method+bl)       psi(region+cover+bl + dwc)" = occu(~region+method+bl     ~region+cover+bl+dwc,
grey.umf),
"p(region+method+dens)     psi(region+cover+bl + dwc)" = occu(~region+method+dens   ~region+cover+bl+dwc,
grey.umf),
"p(region+method+dwc)      psi(region+cover+bl + dwc)" = occu(~region+method+dwc    ~region+cover+bl+dwc,
grey.umf),
"p(region+method+visit)    psi(region+cover+bl + dwc)" = occu(~region+method+visit  ~region+cover+bl+dwc,
grey.umf),
```

```
"p(region+cover+bl)    psi(region+cover+bl + dwc)" = occu(~region+cover+bl    ~region+cover+bl+dwc, grey.umf),
"p(region+cover+dens)    psi(region+cover+bl + dwc)" = occu(~region+cover+dens    ~region+cover+bl+dwc, grey.umf),
"p(region+cover+dwc)    psi(region+cover+bl + dwc)" = occu(~region+cover+dwc    ~region+cover+bl+dwc, grey.umf),
"p(region+cover+visit)    psi(region+cover+bl + dwc)" = occu(~region+cover+visit    ~region+cover+bl+dwc, grey.umf),
"p(region+bl+dens)    psi(region+cover+bl + dwc)" = occu(~region+bl+dens    ~region+cover+bl+dwc, grey.umf),
"p(region+bl+dwc)    psi(region+cover+bl + dwc)" = occu(~region+bl+dwc    ~region+cover+bl+dwc, grey.umf),
"p(region+bl+visit)    psi(region+cover+bl + dwc)" = occu(~region+bl+visit    ~region+cover+bl+dwc, grey.umf),
"p(region+dens+visit)    psi(region+cover+bl + dwc)" = occu(~region+dens+visit    ~region+cover+bl+dwc, grey.umf),
"p(region+dwc+visit)    psi(region+cover+bl + dwc)" = occu(~region+dwc+visit    ~region+cover+bl+dwc, grey.umf),
"p(method+cover+bl)    psi(region+cover+bl + dwc)" = occu(~method+cover+bl    ~region+cover+bl+dwc, grey.umf),
"p(method+cover+dens)    psi(region+cover+bl + dwc)" = occu(~region+cover+dens    ~region+cover+bl+dwc, grey.umf),
"p(method+cover+dwc)    psi(region+cover+bl + dwc)" = occu(~method+cover+dwc    ~region+cover+bl+dwc, grey.umf),
"p(method+cover+visit)    psi(region+cover+bl + dwc)" = occu(~method+cover+visit    ~region+cover+bl+dwc, grey.umf),
"p(method+bl+dens)    psi(region+cover+bl + dwc)" = occu(~method+bl+dens    ~region+cover+bl+dwc, grey.umf),
"p(method+bl+dwc)    psi(region+cover+bl + dwc)" = occu(~method+bl+dwc    ~region+cover+bl+dwc, grey.umf),
"p(method+bl+visit)    psi(region+cover+bl + dwc)" = occu(~method+bl+visit    ~region+cover+bl+dwc, grey.umf),
"p(method+dens+visit)    psi(region+cover+bl + dwc)" = occu(~method+dens+visit    ~region+cover+bl+dwc, grey.umf),
"p(method+dwc+visit)    psi(region+cover+bl + dwc)" = occu(~method+dwc+visit    ~region+cover+bl+dwc, grey.umf),
"p(cover+bl+dens)    psi(region+cover+bl + dwc)" = occu(~cover+bl+dens    ~region+cover+bl+dwc, grey.umf),
"p(cover+bl+dwc)    psi(region+cover+bl + dwc)" = occu(~cover+bl+dwc    ~region+cover+bl+dwc, grey.umf),
"p(cover+bl+visit)    psi(region+cover+bl + dwc)" = occu(~cover+bl+visit    ~region+cover+bl+dwc, grey.umf),
"p(cover+dens+visit)    psi(region+cover+bl + dwc)" = occu(~cover+dens+visit    ~region+cover+bl+dwc, grey.umf),
"p(cover+dwc+visit)    psi(region+cover+bl + dwc)" = occu(~cover+dwc+visit    ~region+cover+bl+dwc, grey.umf),
"p(bl+dens+visit)    psi(region+cover+bl + dwc)" = occu(~bl+dens+visit    ~region+cover+bl+dwc, grey.umf),
"p(bl+dwc+visit)    psi(region+cover+bl + dwc)" = occu(~bl+dwc+visit    ~region+cover+bl+dwc, grey.umf),

#four terms
"p(region+method+cover+bl)    psi(region+cover+bl + dwc)" = occu(~region+method+cover+bl    ~region+cover+bl+dwc, grey.umf),
"p(region+method+cover+dens)    psi(region+cover+bl + dwc)" = occu(~region+method+cover+dens    ~region+cover+bl+dwc, grey.umf),
```

```
  "p(region+method+cover+dwc)         psi(region+cover+bl + dwc)" = occu(~region+method+cover+dwc
~region+cover+bl+dwc, grey.umf),

  "p(region+method+cover+visit)       psi(region+cover+bl + dwc)" = occu(~region+method+cover+visit
~region+cover+bl+dwc, grey.umf),

  "p(region+method+bl+dens)           psi(region+cover+bl + dwc)" = occu(~region+method+bl+dens
~region+cover+bl+dwc, grey.umf),

  "p(region+method+bl+dwc)            psi(region+cover+bl + dwc)" = occu(~region+method+bl+dwc
~region+cover+bl+dwc, grey.umf),

  "p(region+method+bl+visit)          psi(region+cover+bl + dwc)" = occu(~region+method+bl+visit    ~region+cover+bl+dwc,
grey.umf),

  "p(region+method+dens+visit)        psi(region+cover+bl + dwc)" = occu(~region+method+dens+visit
~region+cover+bl+dwc, grey.umf),

  "p(region+method+dwc+visit)         psi(region+cover+bl + dwc)" = occu(~region+method+dwc+visit
~region+cover+bl+dwc, grey.umf),

  "p(region+cover+bl+dens)            psi(region+cover+bl + dwc)" = occu(~region+cover+bl+dens      ~region+cover+bl+dwc,
grey.umf),

  "p(region+cover+bl+dwc)             psi(region+cover+bl + dwc)" = occu(~region+cover+bl+dwc       ~region+cover+bl+dwc,
grey.umf),

  "p(region+cover+bl+visit)           psi(region+cover+bl + dwc)" = occu(~region+cover+bl+visit     ~region+cover+bl+dwc,
grey.umf),

  "p(region+cover+dens+visit)         psi(region+cover+bl + dwc)" = occu(~region+cover+dens+visit   ~region+cover+bl+dwc,
grey.umf),

  "p(region+cover+dwc+visit)          psi(region+cover+bl + dwc)" = occu(~region+cover+dwc+visit    ~region+cover+bl+dwc,
grey.umf),

  "p(region+bl+dens+visit)            psi(region+cover+bl + dwc)" = occu(~region+bl+dens+visit      ~region+cover+bl+dwc,
grey.umf),

  "p(region+bl+dwc+visit)             psi(region+cover+bl + dwc)" = occu(~region+bl+dwc+visit       ~region+cover+bl+dwc,
grey.umf),

  "p(method+cover+bl+dens)            psi(region+cover+bl + dwc)" = occu(~method+cover+bl+dens      ~region+cover+bl+dwc,
grey.umf),

  "p(method+cover+bl+dwc)             psi(region+cover+bl + dwc)" = occu(~method+cover+bl+dwc       ~region+cover+bl+dwc,
grey.umf),

  "p(method+cover+bl+visit)           psi(region+cover+bl + dwc)" = occu(~method+cover+bl+visit     ~region+cover+bl+dwc,
grey.umf),

  "p(method+cover+dens+visit)         psi(region+cover+bl + dwc)" = occu(~method+cover+dens+visit
~region+cover+bl+dwc, grey.umf),

  "p(method+cover+dwc+visit)          psi(region+cover+bl + dwc)" = occu(~method+cover+dwc+visit
~region+cover+bl+dwc, grey.umf),

  "p(method+bl+dens+visit)            psi(region+cover+bl + dwc)" = occu(~method+bl+dens+visit      ~region+cover+bl+dwc,
grey.umf),

  "p(method+bl+dwc+visit)             psi(region+cover+bl + dwc)" = occu(~method+bl+dwc+visit       ~region+cover+bl+dwc,
grey.umf),

  "p(cover+bl+dens+visit)             psi(region+cover+bl + dwc)" = occu(~cover+bl+dens+visit       ~region+cover+bl+dwc,
grey.umf),

  "p(cover+bl+dwc+visit)              psi(region+cover+bl + dwc)" = occu(~cover+bl+dwc+visit        ~region+cover+bl+dwc,
grey.umf),
```

```
  #five terms
  "p(region+method+cover+bl+dens)      psi(region+cover+bl + dwc)" = occu(~region+method+cover+bl+dens
~region+cover+bl+dwc, grey.umf),
  "p(region+method+cover+bl+dwc)       psi(region+cover+bl + dwc)" = occu(~region+method+cover+bl+dwc
~region+cover+bl+dwc, grey.umf),
  "p(region+method+cover+bl+visit)     psi(region+cover+bl + dwc)" = occu(~region+method+cover+bl+visit
~region+cover+bl+dwc, grey.umf),
  "p(region+method+cover+dens+visit)   psi(region+cover+bl + dwc)" = occu(~region+method+cover+dens+visit
~region+cover+bl+dwc, grey.umf),
  "p(region+method+cover+dwc+visit)    psi(region+cover+bl + dwc)" = occu(~region+method+cover+dwc+visit
~region+cover+bl+dwc, grey.umf),
  "p(region+method+bl+dens+visit)      psi(region+cover+bl + dwc)" = occu(~region+method+bl+dens+visit
~region+cover+bl+dwc, grey.umf),
  "p(region+method+bl+dwc+visit)       psi(region+cover+bl + dwc)" = occu(~region+method+bl+dwc+visit
~region+cover+bl+dwc, grey.umf),
  "p(region+cover+bl+dens+visit)       psi(region+cover+bl + dwc)" = occu(~region+cover+bl+dens+visit
~region+cover+bl+dwc, grey.umf),
  "p(region+cover+bl+dwc+visit)        psi(region+cover+bl + dwc)" = occu(~region+cover+bl+dwc+visit
~region+cover+bl+dwc, grey.umf),
  "p(method+cover+bl+dens+visit)       psi(region+cover+bl + dwc)" = occu(~method+cover+bl+dens+visit
~region+cover+bl+dwc, grey.umf),
  "p(method+cover+bl+dwc+visit)        psi(region+cover+bl + dwc)" = occu(~method+cover+bl+dwc+visit
~region+cover+bl+dwc, grey.umf),


  #six terms
  "p(region+method+cover+bl+dens+visit)psi(region+cover+bl + dwc)" =
occu(~region+method+cover+bl+dens+visit~region+cover+bl+dwc, grey.umf),
  "p(region+method+cover+bl+dwc+visit) psi(region+cover+bl + dwc)" = occu(~region+method+cover+bl+dwc+visit
~region+cover+bl+dwc, grey.umf)
)
grey.p.fl <- fitList(fits=grey.p.mods)
grey.p.ms <- modSel(grey.p.fl)
grey.p.ms


### CONCLUSIONS from step 1:
###    - 'region+dwc+visit' is aic best model
###    - method, bl, cover are 'uninformative parameters' (Arnold 2010)
###    - proceed with 'region+dwc+visit'
```

```
####################################################################################
## Stage two: compare occupancy models using a aic-best detection structure

grey.psi.mods <- list(#~p ~psi

 ##single term
 "p(1)                psi(1)"          = occu(~1                ~1,         grey.umf),
 "p(region+dwc+visit)        psi(1)"          = occu(~region+dwc+visit      ~1,         grey.umf),
 "p(region+dwc+visit)        psi(region)"     = occu(~region+dwc+visit      ~region,     grey.umf),
 "p(region+dwc+visit)        psi(dens)"       = occu(~region+dwc+visit      ~dens,       grey.umf),
 "p(region+dwc+visit)        psi(dwc)"        = occu(~region+dwc+visit      ~dwc,        grey.umf),
 "p(region+dwc+visit)        psi(cover)"      = occu(~region+dwc+visit      ~cover,      grey.umf),
 "p(region+dwc+visit)        psi(bl)"         = occu(~region+dwc+visit      ~bl,         grey.umf),

 ##two term inc *interactions
 "p(region+dwc+visit)        psi(region+dens)"    = occu(~region+dwc+visit      ~region+dens,  grey.umf),
 "p(region+dwc+visit)        psi(region*dens)"    = occu(~region+dwc+visit      ~region*dens,  grey.umf),
 "p(region+dwc+visit)        psi(region+dwc)"     = occu(~region+dwc+visit      ~region+dwc,   grey.umf),
 "p(region+dwc+visit)        psi(region*dwc)"     = occu(~region+dwc+visit      ~region*dwc,   grey.umf),
 "p(region+dwc+visit)        psi(region+bl)"      = occu(~region+dwc+visit      ~region+bl,    grey.umf),
 "p(region+dwc+visit)        psi(region*bl)"      = occu(~region+dwc+visit      ~region*cover, grey.umf),
 "p(region+dwc+visit)        psi(region+cover)"   = occu(~region+dwc+visit      ~region+cover, grey.umf),
 "p(region+dwc+visit)        psi(region*cover)"   = occu(~region+dwc+visit      ~region*cover, grey.umf),
 "p(region+dwc+visit)        psi(bl+cover)"       = occu(~region+dwc+visit      ~bl+cover,     grey.umf),
 "p(region+dwc+visit)        psi(bl+dens)"        = occu(~region+dwc+visit      ~bl+dens,      grey.umf),
 "p(region+dwc+visit)        psi(bl+dwc)"         = occu(~region+dwc+visit      ~bl+dwc,       grey.umf),
 "p(region+dwc+visit)        psi(dwc*bl)"         = occu(~region+dwc+visit      ~dwc*bl,       grey.umf),
 "p(region+dwc+visit)        psi(dens*bl)"        = occu(~region+dwc+visit      ~dens*bl,      grey.umf),
 "p(region+dwc+visit)        psi(cover+dens)"     = occu(~region+dwc+visit      ~cover+dens,   grey.umf),
 "p(region+dwc+visit)        psi(cover+dwc)"      = occu(~region+dwc+visit      ~cover+dwc,    grey.umf),
 "p(region+dwc+visit)        psi(cover*dens)"     = occu(~region+dwc+visit      ~cover*dens,   grey.umf),
 "p(region+dwc+visit)        psi(cover*dwc)"      = occu(~region+dwc+visit      ~cover*dwc,    grey.umf),

 ##three term w/o interactions
 "p(region+dwc+visit)        psi(region+bl+cover)"  = occu(~region+dwc+visit      ~region+bl+cover,   grey.umf),
```

```
"p(region+dwc+visit)            psi(region+bl+dens)"   = occu(~region+dwc+visit          ~region+bl+dens,    grey.umf),
"p(region+dwc+visit)            psi(region+bl+dwc)"    = occu(~region+dwc+visit          ~region+bl+dwc,     grey.umf),
"p(region+dwc+visit)            psi(region+cover+dens)"= occu(~region+dwc+visit          ~region+cover+dens, grey.umf),
"p(region+dwc+visit)            psi(region+cover+dwc)" = occu(~region+dwc+visit          ~region+cover+dwc,  grey.umf),
"p(region+dwc+visit)            psi(bl+cover+dens)"    = occu(~region+dwc+visit          ~bl+cover+dens,     grey.umf),
"p(region+dwc+visit)            psi(bl+cover+dwc)"     = occu(~region+dwc+visit          ~bl+cover+dwc,      grey.umf),

##four term w/o interactions
"p(region+dwc+visit)            psi(region+bl+cover+dens)" = occu(~region+dwc+visit     ~region+bl+cover+dens, grey.umf),
"p(region+dwc+visit)            psi(region+bl+cover+dwc)"  =occu(~region+dwc+visit      ~region+bl+cover+dwc,  grey.umf),

##three term with interactions
"p(region+dwc+visit)     psi(region*dwc+cover)"        =occu(~region+dwc+visit   ~region*dwc+cover,   grey.umf),
"p(region+dwc+visit)     psi(region*dwc+bl)"           =occu(~region+dwc+visit   ~region*dwc+bl,      grey.umf),
"p(region+dwc+visit)     psi(region*dens+cover)"       =occu(~region+dwc+visit   ~region*dens+cover,  grey.umf),
"p(region+dwc+visit)     psi(region*dens+bl)"          =occu(~region+dwc+visit   ~region*dens+bl,     grey.umf),
"p(region+dwc+visit)     psi(region*cover+dwc)"        =occu(~region+dwc+visit   ~region*cover+dwc,   grey.umf),
"p(region+dwc+visit)     psi(region*cover+dens)"       =occu(~region+dwc+visit   ~region*cover+dens,  grey.umf),
"p(region+dwc+visit)     psi(region*cover+bl)"         =occu(~region+dwc+visit   ~region*cover+bl,    grey.umf),
"p(region+dwc+visit)     psi(region*bl+dwc)"           =occu(~region+dwc+visit   ~region*bl+dwc,      grey.umf),
"p(region+dwc+visit)     psi(region*bl+dens)"          =occu(~region+dwc+visit   ~region*bl+dens,     grey.umf),
"p(region+dwc+visit)     psi(region*bl+cover)"         =occu(~region+dwc+visit   ~region*bl+cover,    grey.umf),
"p(region+dwc+visit)     psi(dwc*bl+region)"           =occu(~region+dwc+visit   ~dwc*bl+region,      grey.umf),
"p(region+dwc+visit)     psi(dwc*bl+cover)"            =occu(~region+dwc+visit   ~dwc*bl+cover,       grey.umf),
"p(region+dwc+visit)     psi(dens*bl+region)"          =occu(~region+dwc+visit   ~dens*bl+region,     grey.umf),
"p(region+dwc+visit)     psi(dens*bl+cover)"           =occu(~region+dwc+visit   ~dens*bl+cover,      grey.umf),
"p(region+dwc+visit)     psi(dwc*cover+region)"        =occu(~region+dwc+visit   ~dwc*cover+region,   grey.umf),
"p(region+dwc+visit)     psi(dwc*cover+bl)"            =occu(~region+dwc+visit   ~dwc*cover+bl,       grey.umf),
"p(region+dwc+visit)     psi(dens*cover+region)"       =occu(~region+dwc+visit   ~dens*cover+region,  grey.umf),
"p(region+dwc+visit)     psi(dens*cover+bl)"           =occu(~region+dwc+visit   ~dens*cover+bl,      grey.umf),

##four term inc interactions:
"p(region+dwc+visit)     psi(region*dwc+cover+bl)"      =occu(~region+dwc+visit    ~region*dwc+cover+bl,  grey.umf),
"p(region+dwc+visit)     psi(region*dens+cover+bl)"     =occu(~region+dwc+visit    ~region*dens+cover+bl, grey.umf),
"p(region+dwc+visit)     psi(region*cover+dwc+bl)"      =occu(~region+dwc+visit    ~region*cover+dwc+bl,  grey.umf),
"p(region+dwc+visit)     psi(region*cover+dens+bl)"     =occu(~region+dwc+visit    ~region*cover+dens+bl, grey.umf),
"p(region+dwc+visit)     psi(region*bl+dwc+cover)"      =occu(~region+dwc+visit    ~region*bl+dwc+cover,  grey.umf),
```

```
  "p(region+dwc+visit)    psi(region*bl+dens+cover)"    =occu(~region+dwc+visit   ~region*bl+dens+cover, grey.umf),
  "p(region+dwc+visit)    psi(dwc*bl+region+cover)"     =occu(~region+dwc+visit   ~dwc*bl+region+cover,  grey.umf),
  "p(region+dwc+visit)    psi(dens*bl+region+cover)"    =occu(~region+dwc+visit   ~dens*bl+region+cover, grey.umf),
  "p(region+dwc+visit)    psi(dwc*cover+region+bl)"     =occu(~region+dwc+visit   ~dwc*cover+region+bl,  grey.umf),
  "p(region+dwc+visit)    psi(dens*cover+region+bl)"    =occu(~region+dwc+visit   ~dens*cover+region+bl, grey.umf)
)

grey.psi.fl <- fitList(fits=grey.psi.mods)
grey.psi.ms <- modSel(grey.psi.fl)
grey.psi.ms

### CONCLUSIONS from step 2:
###    - region*bl+dwc basically the aic-top, and clear top model
###    - cover is an 'uninformative parameter' (Arnold 2010)
###    - some model uncertainty so model do average predictions


####################################################
# Coefficient table

coef.tab <- grey.psi.ms@Full
write.csv(coef.tab, file="grey_coef_tab.csv")




####################################################
# Model average predictions

# occupancy ~ dwc [holding bl, cover and dens @ median]

####################################################
# model averaged predictions: GS ~ dwc

nn <- 500

#set up the covariates for the wc partial regression
region <- rep(c("BO","CS"), each=nn)
```

```
dwc <- c(with(grey[grey$region %in% "BO",],seq(min(dwc),max(dwc),length=nn)),
        with(grey[grey$region %in% "CS",],seq(min(dwc),max(dwc),length=nn)))

bl <- c(with(grey[grey$region %in% "BO",],seq(min(bl),max(bl),length=nn)),
       with(grey[grey$region %in% "CS",],seq(min(bl),max(bl),length=nn)))

dens <- c(with(grey[grey$region %in% "BO",],seq(min(dens),max(dens),length=nn)),
         with(grey[grey$region %in% "CS",],seq(min(dens),max(dens),length=nn)))

cover <- c(with(grey[grey$region %in% "BO",],seq(min(cover),max(cover),length=nn)),
          with(grey[grey$region %in% "CS",],seq(min(cover),max(cover),length=nn)))


for(j in 1:2){
mid_metric <- c("mean","median")[j]
BO.val <- apply(grey[grey$region%in%"BO",c("bl","cover","dens","dwc")],2,mid_metric)
CS.val <- apply(grey[grey$region%in%"CS",c("bl","cover","dens","dwc")],2,mid_metric)

bl.mu    <- c(rep(BO.val[1], nn), rep(CS.val[1], nn))
cover.mu <- c(rep(BO.val[2], nn), rep(CS.val[2], nn))
dens.mu  <- c(rep(BO.val[3], nn), rep(CS.val[3], nn))
dwc.mu   <- c(rep(BO.val[4], nn), rep(CS.val[4], nn))

new.df.g <- data.frame(region = region,
                    bl     = bl.mu,      #mean bl
                    dens   = dens.mu,    #mean dens
                    cover  = cover.mu,   #mean cover
                    dwc    = dwc)        #vary dwc

pred.dwc.g <- predict(grey.psi.fl, type="state", newdata = new.df.g)

# Plot - can decide on type and resolution here too:
for(i in 1:3){
  if(i == 1) pdf(paste0("grey_by_dwc.se.",mid_metric,".pdf"), height=6,     width=6)
  if(i == 2) png(paste0("grey_by_dwc.se.",mid_metric,".png"), height = 500, width = 500, pointsize = 16, res=72)
  if(i == 3) tiff(paste0("grey_by_dwc.se.",mid_metric,".tif"),height = 500, width = 500, pointsize = 16, res=72)
  plot(new.df.g$dwc, new.df.g$dwc, ylim=c(0,1), type="n", xlab="Pine Marten Connectivity",
```

```
     ylab="Occupancy", bty="l", las=1, xlim=c(0,3))
  polygon(c(new.df.g$dwc[1:nn],rev(new.df.g$dwc[1:nn])),
        c(pred.dwc.g[1:nn,1]+pred.dwc.g[1:nn,2],rev(pred.dwc.g[1:nn,1]-pred.dwc.g[1:nn,2])),
        border=F,col=adjustcolor("darkblue",0.5))
  polygon(c(new.df.g$dwc[(nn+1):(nn*2)],rev(new.df.g$dwc[(nn+1):(nn*2)])),
        c(pred.dwc.g[(nn+1):(nn*2),1]+pred.dwc.g[(nn+1):(nn*2),2],
          rev(pred.dwc.g[(nn+1):(nn*2),1]-pred.dwc.g[(nn+1):(nn*2),2])),
        border=F,col=adjustcolor("darkgreen",0.5))
  lines(new.df.g$dwc[1:nn],pred.dwc.g[1:nn,1],lwd=2)
  lines(new.df.g$dwc[(nn+1):(nn*2)],pred.dwc.g[(nn+1):(nn*2),1],lwd=2)
  legend("topright",c("Borders","Central"),pch=15, bty="n",
       col=adjustcolor(c("darkblue","darkgreen")),cex=1.2)
  dev.off()
}
}
```
