# Supplementary material for: The enemy of my enemy is my friend: native pine marten recovery reverses the decline of the red squirrel by suppressing grey squirrel populations
Source: Proc Biol Sci. 2018 Mar 7;285(1874):20172603. doi: 10.1098/rspb.2017.2603 (PMC5879625; doi:10.1098/rspb.2017.2603)
Supplement: File S4 from The enemy of my enemy is my friend: Native pine marten recovery reverses the decline of the red squirrel by suppressing grey squirrel populations [file rspb20172603supp4.pdf]

```
library(unmarked)
library(raster)

setwd()
load("aveDensity.Rdata")    # output file from scr, i.e. need to have run scr analysis first
load("aveDWC.Rdata")        # also scr ouptput file

detections <-   read.csv("detections_red.csv")
red  <- subset(detections, Species %in% "red")

tmp1 <- cbind(extract(aveDensity[[1]],as.matrix(red[,c("X","Y")]/1000)),
            extract(aveDensity[[2]],as.matrix(red[,c("X","Y")]/1000)),
            extract(aveDensity[[3]],as.matrix(red[,c("X","Y")]/1000)))
tmp2 <- cbind(extract(aveDWC[[1]],    as.matrix(red[,c("X","Y")]/1000)),
            extract(aveDWC[[2]],    as.matrix(red[,c("X","Y")]/1000)),
            extract(aveDWC[[3]],    as.matrix(red[,c("X","Y")]/1000)))

red$dens  <- apply(tmp1,1,sum,na.rm=T) * 1/(0.1^2)
red$dwc  <- apply(tmp2,1,sum,na.rm=T)

rmv <- which(apply(is.na(red[,paste0("visit",1:5)]),1,sum)%in%5)

obsCovs <- list()
obsCovs[["method"]] <- red[-rmv,paste0("tCov",1:5)]
obsCovs[["visit"]]  <- matrix(factor(col(obsCovs[[1]])),
                         nrow(obsCovs[[1]]),ncol(obsCovs[[1]]))
colnames(obsCovs[["method"]]) <- paste0("method.",1:5)
colnames(obsCovs[["visit"]])  <- paste0("visit.", 1:5)
red.umf <- unmarkedFrameOccu(y=red[-rmv,paste0("visit",1:5)],
                        siteCovs = red[-rmv,c("region","dens", "dwc", "cover", "bl", "gs")],
                        obsCovs = obsCovs)
```

```
#######################################################################################
## Stage one: compare detection models using a flexible occupancy structure
red.p.mods <- list(#~p


#psi~.+density
  #single terms
  "p(1)                          psi(region+cover+bl+gs + dens)" = occu(~1       ~region+cover+bl+gs+dens, red.umf),
  "p(region)                     psi(region+cover+bl+gs + dens)" = occu(~region ~region+cover+bl+gs+dens, red.umf),
  "p(method)                     psi(region+cover+bl+gs + dens)" = occu(~method ~region+cover+bl+gs+dens, red.umf),
  "p(cover)                      psi(region+cover+bl+gs + dens)" = occu(~cover ~region+cover+bl+gs+dens, red.umf),
  "p(bl)                         psi(region+cover+bl+gs + dens)" = occu(~bl      ~region+cover+bl+gs+dens, red.umf),
  "p(dens)                       psi(region+cover+bl+gs + dens)" = occu(~dens ~region+cover+bl+gs+dens, red.umf),
  "p(dwc)                        psi(region+cover+bl+gs + dens)" = occu(~dwc     ~region+cover+bl+gs+dens, red.umf),
  "p(visit)                      psi(region+cover+bl+gs + dens)" = occu(~visit   ~region+cover+bl+gs+dens, red.umf),


  #two terms
  "p(region+method)              psi(region+cover+bl+gs + dens)" = occu(~region+method ~region+cover+bl+gs+dens, red.umf),
  "p(region+cover)               psi(region+cover+bl+gs + dens)" = occu(~region+cover ~region+cover+bl+gs+dens, red.umf),
  "p(region+bl)                  psi(region+cover+bl+gs + dens)" = occu(~region+bl ~region+cover+bl+gs+dens, red.umf),
  "p(region+dens)                psi(region+cover+bl+gs + dens)" = occu(~region+dens ~region+cover+bl+gs+dens, red.umf),
  "p(region+dwc)                 psi(region+cover+bl+gs + dens)" = occu(~region+dwc ~region+cover+bl+gs+dens, red.umf),
```

```
  "p(region+visit)                    psi(region+cover+bl+gs + dens)" = occu(~region+visit
~region+cover+bl+gs+dens, red.umf),

  "p(method+cover)                    psi(region+cover+bl+gs + dens)" = occu(~method+cover
~region+cover+bl+gs+dens, red.umf),

  "p(method+bl)                       psi(region+cover+bl+gs + dens)" = occu(~method+bl
~region+cover+bl+gs+dens, red.umf),

  "p(method+dens)                     psi(region+cover+bl+gs + dens)" = occu(~method+dens
~region+cover+bl+gs+dens, red.umf),

  "p(method+dwc)                      psi(region+cover+bl+gs + dens)" = occu(~method+dwc
~region+cover+bl+gs+dens, red.umf),

  "p(method+visit)                    psi(region+cover+bl+gs + dens)" = occu(~method+visit
~region+cover+bl+gs+dens, red.umf),

  "p(cover+bl)                        psi(region+cover+bl+gs + dens)" = occu(~cover+bl
~region+cover+bl+gs+dens, red.umf),

  "p(cover+dens)                      psi(region+cover+bl+gs + dens)" = occu(~cover+dens
~region+cover+bl+gs+dens, red.umf),

  "p(cover+dwc)                       psi(region+cover+bl+gs + dens)" = occu(~cover+dwc
~region+cover+bl+gs+dens, red.umf),

  "p(cover+visit)                     psi(region+cover+bl+gs + dens)" = occu(~cover+visit
~region+cover+bl+gs+dens, red.umf),

  "p(bl+dens)                         psi(region+cover+bl+gs + dens)" = occu(~bl+dens
~region+cover+bl+gs+dens, red.umf),

  "p(bl+dwc)                          psi(region+cover+bl+gs + dens)" = occu(~bl+dwc
~region+cover+bl+gs+dens, red.umf),

  "p(bl+visit)                        psi(region+cover+bl+gs + dens)" = occu(~bl+visit
~region+cover+bl+gs+dens, red.umf),

  "p(dens+visit)                      psi(region+cover+bl+gs + dens)" = occu(~dens+visit
~region+cover+bl+gs+dens, red.umf),

  "p(dwc+visit)                       psi(region+cover+bl+gs + dens)" = occu(~dwc+visit
~region+cover+bl+gs+dens, red.umf),


  #three terms

  "p(region+method+cover)             psi(region+cover+bl+gs + dens)" = occu(~region+method+cover
~region+cover+bl+gs+dens, red.umf),

  "p(region+method+bl)                psi(region+cover+bl+gs + dens)" = occu(~region+method+bl
~region+cover+bl+gs+dens, red.umf),
```

```
  "p(region+method+dens)             psi(region+cover+bl+gs + dens)" = occu(~region+method+dens
~region+cover+bl+gs+dens, red.umf),

  "p(region+method+dwc)              psi(region+cover+bl+gs + dens)" = occu(~region+method+dwc
~region+cover+bl+gs+dens, red.umf),

  "p(region+method+visit)            psi(region+cover+bl+gs + dens)" = occu(~region+method+visit
~region+cover+bl+gs+dens, red.umf),

  "p(region+cover+bl)                psi(region+cover+bl+gs + dens)" = occu(~region+cover+bl
~region+cover+bl+gs+dens, red.umf),

  "p(region+cover+dens)              psi(region+cover+bl+gs + dens)" = occu(~region+cover+dens
~region+cover+bl+gs+dens, red.umf),

  "p(region+cover+dwc)               psi(region+cover+bl+gs + dens)" = occu(~region+cover+dwc
~region+cover+bl+gs+dens, red.umf),

  "p(region+cover+visit)             psi(region+cover+bl+gs + dens)" = occu(~region+cover+visit
~region+cover+bl+gs+dens, red.umf),

  "p(region+bl+dens)                 psi(region+cover+bl+gs + dens)" = occu(~region+bl+dens
~region+cover+bl+gs+dens, red.umf),

  "p(region+bl+dwc)                  psi(region+cover+bl+gs + dens)" = occu(~region+bl+dwc
~region+cover+bl+gs+dens, red.umf),

  "p(region+bl+visit)                psi(region+cover+bl+gs + dens)" = occu(~region+bl+visit
~region+cover+bl+gs+dens, red.umf),

  "p(region+dens+visit)              psi(region+cover+bl+gs + dens)" = occu(~region+dens+visit
~region+cover+bl+gs+dens, red.umf),

  "p(region+dwc+visit)               psi(region+cover+bl+gs + dens)" = occu(~region+dwc+visit
~region+cover+bl+gs+dens, red.umf),

  "p(method+cover+bl)                psi(region+cover+bl+gs + dens)" = occu(~method+cover+bl
~region+cover+bl+gs+dens, red.umf),

  "p(method+cover+dens)              psi(region+cover+bl+gs + dens)" = occu(~region+cover+dens
~region+cover+bl+gs+dens, red.umf),

  "p(method+cover+dwc)               psi(region+cover+bl+gs + dens)" = occu(~method+cover+dwc
~region+cover+bl+gs+dens, red.umf),

  "p(method+cover+visit)             psi(region+cover+bl+gs + dens)" = occu(~method+cover+visit
~region+cover+bl+gs+dens, red.umf),

  "p(method+bl+dens)                 psi(region+cover+bl+gs + dens)" = occu(~method+bl+dens
~region+cover+bl+gs+dens, red.umf),

  "p(method+bl+dwc)                  psi(region+cover+bl+gs + dens)" = occu(~method+bl+dwc
~region+cover+bl+gs+dens, red.umf),

  "p(method+bl+visit)                psi(region+cover+bl+gs + dens)" = occu(~method+bl+visit
~region+cover+bl+gs+dens, red.umf),
```

"p(method+dens+visit)   psi(region+cover+bl+gs + dens)" = occu(~method+dens+visit ~region+cover+bl+gs+dens, red.umf),

"p(method+dwc+visit)   psi(region+cover+bl+gs + dens)" = occu(~method+dwc+visit ~region+cover+bl+gs+dens, red.umf),

"p(cover+bl+dens)   psi(region+cover+bl+gs + dens)" = occu(~cover+bl+dens ~region+cover+bl+gs+dens, red.umf),

"p(cover+bl+dwc)   psi(region+cover+bl+gs + dens)" = occu(~cover+bl+dwc ~region+cover+bl+gs+dens, red.umf),

"p(cover+bl+visit)   psi(region+cover+bl+gs + dens)" = occu(~cover+bl+visit ~region+cover+bl+gs+dens, red.umf),

"p(cover+dens+visit)   psi(region+cover+bl+gs + dens)" = occu(~cover+dens+visit ~region+cover+bl+gs+dens, red.umf),

"p(cover+dwc+visit)   psi(region+cover+bl+gs + dens)" = occu(~cover+dwc+visit ~region+cover+bl+gs+dens, red.umf),

"p(bl+dens+visit)   psi(region+cover+bl+gs + dens)" = occu(~bl+dens+visit ~region+cover+bl+gs+dens, red.umf),

"p(bl+dwc+visit)   psi(region+cover+bl+gs + dens)" = occu(~bl+dwc+visit ~region+cover+bl+gs+dens, red.umf),

#four terms

"p(region+method+cover+bl)   psi(region+cover+bl+gs + dens)" = occu(~region+method+cover+bl   ~region+cover+bl+gs+dens, red.umf),

"p(region+method+cover+dens)   psi(region+cover+bl+gs + dens)" = occu(~region+method+cover+dens ~region+cover+bl+gs+dens, red.umf),

"p(region+method+cover+dwc)   psi(region+cover+bl+gs + dens)" = occu(~region+method+cover+dwc   ~region+cover+bl+gs+dens, red.umf),

"p(region+method+cover+visit)   psi(region+cover+bl+gs + dens)" = occu(~region+method+cover+visit ~region+cover+bl+gs+dens, red.umf),

"p(region+method+bl+dens)   psi(region+cover+bl+gs + dens)" = occu(~region+method+bl+dens   ~region+cover+bl+gs+dens, red.umf),

"p(region+method+bl+dwc)   psi(region+cover+bl+gs + dens)" = occu(~region+method+bl+dwc ~region+cover+bl+gs+dens, red.umf),

"p(region+method+bl+visit)   psi(region+cover+bl+gs + dens)" = occu(~region+method+bl+visit ~region+cover+bl+gs+dens, red.umf),

"p(region+method+dens+visit)   psi(region+cover+bl+gs + dens)" = occu(~region+method+dens+visit ~region+cover+bl+gs+dens, red.umf),

"p(region+method+dwc+visit)   psi(region+cover+bl+gs + dens)" =
occu(~region+method+dwc+visit   ~region+cover+bl+gs+dens, red.umf),

"p(region+cover+bl+dens)   psi(region+cover+bl+gs + dens)" = occu(~region+cover+bl+dens
~region+cover+bl+gs+dens, red.umf),

"p(region+cover+bl+dwc)   psi(region+cover+bl+gs + dens)" = occu(~region+cover+bl+dwc
~region+cover+bl+gs+dens, red.umf),

"p(region+cover+bl+visit)   psi(region+cover+bl+gs + dens)" = occu(~region+cover+bl+visit
~region+cover+bl+gs+dens, red.umf),

"p(region+cover+dens+visit)   psi(region+cover+bl+gs + dens)" = occu(~region+cover+dens+visit
~region+cover+bl+gs+dens, red.umf),

"p(region+cover+dwc+visit)   psi(region+cover+bl+gs + dens)" = occu(~region+cover+dwc+visit
~region+cover+bl+gs+dens, red.umf),

"p(region+bl+dens+visit)   psi(region+cover+bl+gs + dens)" = occu(~region+bl+dens+visit
~region+cover+bl+gs+dens, red.umf),

"p(region+bl+dwc+visit)   psi(region+cover+bl+gs + dens)" = occu(~region+bl+dwc+visit
~region+cover+bl+gs+dens, red.umf),

"p(method+cover+bl+dens)   psi(region+cover+bl+gs + dens)" = occu(~method+cover+bl+dens
~region+cover+bl+gs+dens, red.umf),

"p(method+cover+bl+dwc)   psi(region+cover+bl+gs + dens)" = occu(~method+cover+bl+dwc
~region+cover+bl+gs+dens, red.umf),

"p(method+cover+bl+visit)   psi(region+cover+bl+gs + dens)" = occu(~method+cover+bl+visit
~region+cover+bl+gs+dens, red.umf),

"p(method+cover+dens+visit)   psi(region+cover+bl+gs + dens)" =
occu(~method+cover+dens+visit   ~region+cover+bl+gs+dens, red.umf),

"p(method+cover+dwc+visit)   psi(region+cover+bl+gs + dens)" =
occu(~method+cover+dwc+visit   ~region+cover+bl+gs+dens, red.umf),

"p(method+bl+dens+visit)   psi(region+cover+bl+gs + dens)" = occu(~method+bl+dens+visit
~region+cover+bl+gs+dens, red.umf),

"p(method+bl+dwc+visit)   psi(region+cover+bl+gs + dens)" = occu(~method+bl+dwc+visit
~region+cover+bl+gs+dens, red.umf),

"p(cover+bl+dens+visit)   psi(region+cover+bl+gs + dens)" = occu(~cover+bl+dens+visit
~region+cover+bl+gs+dens, red.umf),

"p(cover+bl+dwc+visit)   psi(region+cover+bl+gs + dens)" = occu(~cover+bl+dwc+visit
~region+cover+bl+gs+dens, red.umf),

#five terms

"p(region+method+cover+bl+dens)   psi(region+cover+bl+gs + dens)" = occu(~region+method+cover+bl+dens   ~region+cover+bl+gs+dens, red.umf),

"p(region+method+cover+bl+dwc)   psi(region+cover+bl+gs + dens)" = occu(~region+method+cover+bl+dwc   ~region+cover+bl+gs+dens, red.umf),

"p(region+method+cover+bl+visit)   psi(region+cover+bl+gs + dens)" = occu(~region+method+cover+bl+visit   ~region+cover+bl+gs+dens, red.umf),

"p(region+method+cover+dens+visit)   psi(region+cover+bl+gs + dens)" = occu(~region+method+cover+dens+visit ~region+cover+bl+gs+dens, red.umf),

"p(region+method+cover+dwc+visit)   psi(region+cover+bl+gs + dens)" = occu(~region+method+cover+dwc+visit ~region+cover+bl+gs+dens, red.umf),

"p(region+method+bl+dens+visit)   psi(region+cover+bl+gs + dens)" = occu(~region+method+bl+dens+visit   ~region+cover+bl+gs+dens, red.umf),

"p(region+method+bl+dwc+visit)   psi(region+cover+bl+gs + dens)" = occu(~region+method+bl+dwc+visit   ~region+cover+bl+gs+dens, red.umf),

"p(region+cover+bl+dens+visit)   psi(region+cover+bl+gs + dens)" = occu(~region+cover+bl+dens+visit   ~region+cover+bl+gs+dens, red.umf),

"p(region+cover+bl+dwc+visit)   psi(region+cover+bl+gs + dens)" = occu(~region+cover+bl+dwc+visit   ~region+cover+bl+gs+dens, red.umf),

"p(method+cover+bl+dens+visit)   psi(region+cover+bl+gs + dens)" = occu(~method+cover+bl+dens+visit   ~region+cover+bl+gs+dens, red.umf),

"p(method+cover+bl+dwc+visit)   psi(region+cover+bl+gs + dens)" = occu(~method+cover+bl+dwc+visit   ~region+cover+bl+gs+dens, red.umf),

#six terms

"p(region+method+cover+bl+dens+visit)psi(region+cover+bl+gs + dens)" = occu(~region+method+cover+bl+dens+visit~region+cover+bl+gs+dens, red.umf),

"p(region+method+cover+bl+dwc+visit) psi(region+cover+bl+gs + dens)" = occu(~region+method+cover+bl+dwc+visit ~region+cover+bl+gs+dens, red.umf),

DWC

#single terms

"p(1)   psi(region+cover+bl+gs + dwc)" = occu(~1   ~region+cover+bl+gs+dwc, red.umf),

"p(region)   psi(region+cover+bl+gs + dwc)" = occu(~region ~region+cover+bl+gs+dwc, red.umf),

"p(method)   psi(region+cover+bl+gs + dwc)" = occu(~method ~region+cover+bl+gs+dwc, red.umf),

"p(cover)   psi(region+cover+bl+gs + dwc)" = occu(~cover   ~region+cover+bl+gs+dwc, red.umf),

"p(bl)   psi(region+cover+bl+gs + dwc)" = occu(~bl   ~region+cover+bl+gs+dwc, red.umf),

"p(dens)   psi(region+cover+bl+gs + dwc)" = occu(~dens   ~region+cover+bl+gs+dwc, red.umf),

"p(dwc)   psi(region+cover+bl+gs + dwc)" = occu(~dwc   ~region+cover+bl+gs+dwc, red.umf),

"p(visit)   psi(region+cover+bl+gs + dwc)" = occu(~visit   ~region+cover+bl+gs+dwc, red.umf),

#two terms

"p(region+method)   psi(region+cover+bl+gs + dwc)" = occu(~region+method ~region+cover+bl+gs+dwc, red.umf),

"p(region+cover)   psi(region+cover+bl+gs + dwc)" = occu(~region+cover ~region+cover+bl+gs+dwc, red.umf),

"p(region+bl)   psi(region+cover+bl+gs + dwc)" = occu(~region+bl ~region+cover+bl+gs+dwc, red.umf),

"p(region+dens)   psi(region+cover+bl+gs + dwc)" = occu(~region+dens ~region+cover+bl+gs+dwc, red.umf),

"p(region+dwc)   psi(region+cover+bl+gs + dwc)" = occu(~region+dwc ~region+cover+bl+gs+dwc, red.umf),

"p(region+visit)   psi(region+cover+bl+gs + dwc)" = occu(~region+visit ~region+cover+bl+gs+dwc, red.umf),

"p(method+cover)   psi(region+cover+bl+gs + dwc)" = occu(~method+cover ~region+cover+bl+gs+dwc, red.umf),

"p(method+bl)   psi(region+cover+bl+gs + dwc)" = occu(~method+bl ~region+cover+bl+gs+dwc, red.umf),

"p(method+dens)   psi(region+cover+bl+gs + dwc)" = occu(~method+dens ~region+cover+bl+gs+dwc, red.umf),

"p(method+dwc)   psi(region+cover+bl+gs + dwc)" = occu(~method+dwc ~region+cover+bl+gs+dwc, red.umf),

"p(method+visit)   psi(region+cover+bl+gs + dwc)" = occu(~method+visit ~region+cover+bl+gs+dwc, red.umf),

"p(cover+bl)   psi(region+cover+bl+gs + dwc)" = occu(~cover+bl ~region+cover+bl+gs+dwc, red.umf),

```
  "p(cover+dens)                 psi(region+cover+bl+gs + dwc)" = occu(~cover+dens ~region+cover+bl+gs+dwc, red.umf),

  "p(cover+dwc)                  psi(region+cover+bl+gs + dwc)" = occu(~cover+dwc ~region+cover+bl+gs+dwc, red.umf),

  "p(cover+visit)                psi(region+cover+bl+gs + dwc)" = occu(~cover+visit ~region+cover+bl+gs+dwc, red.umf),

  "p(bl+dens)                    psi(region+cover+bl+gs + dwc)" = occu(~bl+dens ~region+cover+bl+gs+dwc, red.umf),

  "p(bl+dwc)                     psi(region+cover+bl+gs + dwc)" = occu(~bl+dwc ~region+cover+bl+gs+dwc, red.umf),

  "p(bl+visit)                   psi(region+cover+bl+gs + dwc)" = occu(~bl+visit ~region+cover+bl+gs+dwc, red.umf),

  "p(dens+visit)                 psi(region+cover+bl+gs + dwc)" = occu(~dens+visit ~region+cover+bl+gs+dwc, red.umf),

  "p(dwc+visit)                  psi(region+cover+bl+gs + dwc)" = occu(~dwc+visit ~region+cover+bl+gs+dwc, red.umf),


  #three terms

  "p(region+method+cover)        psi(region+cover+bl+gs + dwc)" = occu(~region+method+cover ~region+cover+bl+gs+dwc, red.umf),

  "p(region+method+bl)           psi(region+cover+bl+gs + dwc)" = occu(~region+method+bl ~region+cover+bl+gs+dwc, red.umf),

  "p(region+method+dens)         psi(region+cover+bl+gs + dwc)" = occu(~region+method+dens ~region+cover+bl+gs+dwc, red.umf),

  "p(region+method+dwc)          psi(region+cover+bl+gs + dwc)" = occu(~region+method+dwc ~region+cover+bl+gs+dwc, red.umf),

  "p(region+method+visit)        psi(region+cover+bl+gs + dwc)" = occu(~region+method+visit ~region+cover+bl+gs+dwc, red.umf),

  "p(region+cover+bl)            psi(region+cover+bl+gs + dwc)" = occu(~region+cover+bl ~region+cover+bl+gs+dwc, red.umf),

  "p(region+cover+dens)          psi(region+cover+bl+gs + dwc)" = occu(~region+cover+dens ~region+cover+bl+gs+dwc, red.umf),

  "p(region+cover+dwc)           psi(region+cover+bl+gs + dwc)" = occu(~region+cover+dwc ~region+cover+bl+gs+dwc, red.umf),

  "p(region+cover+visit)         psi(region+cover+bl+gs + dwc)" = occu(~region+cover+visit ~region+cover+bl+gs+dwc, red.umf),
```

```
  "p(region+bl+dens)                psi(region+cover+bl+gs + dwc)" = occu(~region+bl+dens
~region+cover+bl+gs+dwc, red.umf),

  "p(region+bl+dwc)                 psi(region+cover+bl+gs + dwc)" = occu(~region+bl+dwc
~region+cover+bl+gs+dwc, red.umf),

  "p(region+bl+visit)               psi(region+cover+bl+gs + dwc)" = occu(~region+bl+visit
~region+cover+bl+gs+dwc, red.umf),

  "p(region+dens+visit)             psi(region+cover+bl+gs + dwc)" = occu(~region+dens+visit
~region+cover+bl+gs+dwc, red.umf),

  "p(region+dwc+visit)              psi(region+cover+bl+gs + dwc)" = occu(~region+dwc+visit
~region+cover+bl+gs+dwc, red.umf),

  "p(method+cover+bl)               psi(region+cover+bl+gs + dwc)" = occu(~method+cover+bl
~region+cover+bl+gs+dwc, red.umf),

  "p(method+cover+dens)             psi(region+cover+bl+gs + dwc)" = occu(~region+cover+dens
~region+cover+bl+gs+dwc, red.umf),

  "p(method+cover+dwc)              psi(region+cover+bl+gs + dwc)" = occu(~method+cover+dwc
~region+cover+bl+gs+dwc, red.umf),

  "p(method+cover+visit)            psi(region+cover+bl+gs + dwc)" = occu(~method+cover+visit
~region+cover+bl+gs+dwc, red.umf),

  "p(method+bl+dens)                psi(region+cover+bl+gs + dwc)" = occu(~method+bl+dens
~region+cover+bl+gs+dwc, red.umf),

  "p(method+bl+dwc)                 psi(region+cover+bl+gs + dwc)" = occu(~method+bl+dwc
~region+cover+bl+gs+dwc, red.umf),

  "p(method+bl+visit)               psi(region+cover+bl+gs + dwc)" = occu(~method+bl+visit
~region+cover+bl+gs+dwc, red.umf),

  "p(method+dens+visit)             psi(region+cover+bl+gs + dwc)" = occu(~method+dens+visit
~region+cover+bl+gs+dwc, red.umf),

  "p(method+dwc+visit)              psi(region+cover+bl+gs + dwc)" = occu(~method+dwc+visit
~region+cover+bl+gs+dwc, red.umf),

  "p(cover+bl+dens)                 psi(region+cover+bl+gs + dwc)" = occu(~cover+bl+dens
~region+cover+bl+gs+dwc, red.umf),

  "p(cover+bl+dwc)                  psi(region+cover+bl+gs + dwc)" = occu(~cover+bl+dwc
~region+cover+bl+gs+dwc, red.umf),

  "p(cover+bl+visit)                psi(region+cover+bl+gs + dwc)" = occu(~cover+bl+visit
~region+cover+bl+gs+dwc, red.umf),

  "p(cover+dens+visit)              psi(region+cover+bl+gs + dwc)" = occu(~cover+dens+visit
~region+cover+bl+gs+dwc, red.umf),

  "p(cover+dwc+visit)               psi(region+cover+bl+gs + dwc)" = occu(~cover+dwc+visit
~region+cover+bl+gs+dwc, red.umf),
```

"p(bl+dens+visit)   psi(region+cover+bl+gs + dwc)" = occu(~bl+dens+visit ~region+cover+bl+gs+dwc, red.umf),

"p(bl+dwc+visit)   psi(region+cover+bl+gs + dwc)" = occu(~bl+dwc+visit ~region+cover+bl+gs+dwc, red.umf),

#four terms

"p(region+method+cover+bl)   psi(region+cover+bl+gs + dwc)" = occu(~region+method+cover+bl   ~region+cover+bl+gs+dwc, red.umf),

"p(region+method+cover+dens)   psi(region+cover+bl+gs + dwc)" = occu(~region+method+cover+dens ~region+cover+bl+gs+dwc, red.umf),

"p(region+method+cover+dwc)   psi(region+cover+bl+gs + dwc)" = occu(~region+method+cover+dwc   ~region+cover+bl+gs+dwc, red.umf),

"p(region+method+cover+visit)   psi(region+cover+bl+gs + dwc)" = occu(~region+method+cover+visit ~region+cover+bl+gs+dwc, red.umf),

"p(region+method+bl+dens)   psi(region+cover+bl+gs + dwc)" = occu(~region+method+bl+dens   ~region+cover+bl+gs+dwc, red.umf),

"p(region+method+bl+dwc)   psi(region+cover+bl+gs + dwc)" = occu(~region+method+bl+dwc ~region+cover+bl+gs+dwc, red.umf),

"p(region+method+bl+visit)   psi(region+cover+bl+gs + dwc)" = occu(~region+method+bl+visit ~region+cover+bl+gs+dwc, red.umf),

"p(region+method+dens+visit)   psi(region+cover+bl+gs + dwc)" = occu(~region+method+dens+visit ~region+cover+bl+gs+dwc, red.umf),

"p(region+method+dwc+visit)   psi(region+cover+bl+gs + dwc)" = occu(~region+method+dwc+visit   ~region+cover+bl+gs+dwc, red.umf),

"p(region+cover+bl+dens)   psi(region+cover+bl+gs + dwc)" = occu(~region+cover+bl+dens ~region+cover+bl+gs+dwc, red.umf),

"p(region+cover+bl+dwc)   psi(region+cover+bl+gs + dwc)" = occu(~region+cover+bl+dwc ~region+cover+bl+gs+dwc, red.umf),

"p(region+cover+bl+visit)   psi(region+cover+bl+gs + dwc)" = occu(~region+cover+bl+visit ~region+cover+bl+gs+dwc, red.umf),

"p(region+cover+dens+visit)   psi(region+cover+bl+gs + dwc)" = occu(~region+cover+dens+visit ~region+cover+bl+gs+dwc, red.umf),

"p(region+cover+dwc+visit)   psi(region+cover+bl+gs + dwc)" = occu(~region+cover+dwc+visit ~region+cover+bl+gs+dwc, red.umf),

"p(region+bl+dens+visit)   psi(region+cover+bl+gs + dwc)" = occu(~region+bl+dens+visit ~region+cover+bl+gs+dwc, red.umf),

```
  "p(region+bl+dwc+visit)            psi(region+cover+bl+gs + dwc)" = occu(~region+bl+dwc+visit
~region+cover+bl+gs+dwc, red.umf),

  "p(method+cover+bl+dens)           psi(region+cover+bl+gs + dwc)" = occu(~method+cover+bl+dens
~region+cover+bl+gs+dwc, red.umf),

  "p(method+cover+bl+dwc)            psi(region+cover+bl+gs + dwc)" = occu(~method+cover+bl+dwc
~region+cover+bl+gs+dwc, red.umf),

  "p(method+cover+bl+visit)          psi(region+cover+bl+gs + dwc)" = occu(~method+cover+bl+visit
~region+cover+bl+gs+dwc, red.umf),

  "p(method+cover+dens+visit)        psi(region+cover+bl+gs + dwc)" =
occu(~method+cover+dens+visit   ~region+cover+bl+gs+dwc, red.umf),

  "p(method+cover+dwc+visit)         psi(region+cover+bl+gs + dwc)" =
occu(~method+cover+dwc+visit    ~region+cover+bl+gs+dwc, red.umf),

  "p(method+bl+dens+visit)           psi(region+cover+bl+gs + dwc)" = occu(~method+bl+dens+visit
~region+cover+bl+gs+dwc, red.umf),

  "p(method+bl+dwc+visit)            psi(region+cover+bl+gs + dwc)" = occu(~method+bl+dwc+visit
~region+cover+bl+gs+dwc, red.umf),

  "p(cover+bl+dens+visit)            psi(region+cover+bl+gs + dwc)" = occu(~cover+bl+dens+visit
~region+cover+bl+gs+dwc, red.umf),

  "p(cover+bl+dwc+visit)             psi(region+cover+bl+gs + dwc)" = occu(~cover+bl+dwc+visit
~region+cover+bl+gs+dwc, red.umf),


  #five terms

  "p(region+method+cover+bl+dens)     psi(region+cover+bl+gs + dwc)" =
occu(~region+method+cover+bl+dens    ~region+cover+bl+gs+dwc, red.umf),

  "p(region+method+cover+bl+dwc)      psi(region+cover+bl+gs + dwc)" =
occu(~region+method+cover+bl+dwc     ~region+cover+bl+gs+dwc, red.umf),

  "p(region+method+cover+bl+visit)    psi(region+cover+bl+gs + dwc)" =
occu(~region+method+cover+bl+visit   ~region+cover+bl+gs+dwc, red.umf),

  "p(region+method+cover+dens+visit)   psi(region+cover+bl+gs + dwc)" =
occu(~region+method+cover+dens+visit ~region+cover+bl+gs+dwc, red.umf),

  "p(region+method+cover+dwc+visit)    psi(region+cover+bl+gs + dwc)" =
occu(~region+method+cover+dwc+visit  ~region+cover+bl+gs+dwc, red.umf),

  "p(region+method+bl+dens+visit)     psi(region+cover+bl+gs + dwc)" =
occu(~region+method+bl+dens+visit    ~region+cover+bl+gs+dwc, red.umf),

  "p(region+method+bl+dwc+visit)      psi(region+cover+bl+gs + dwc)" =
occu(~region+method+bl+dwc+visit     ~region+cover+bl+gs+dwc, red.umf),
```

```
  "p(region+cover+bl+dens+visit)      psi(region+cover+bl+gs + dwc)" =
occu(~region+cover+bl+dens+visit     ~region+cover+bl+gs+dwc, red.umf),

  "p(region+cover+bl+dwc+visit)       psi(region+cover+bl+gs + dwc)" =
occu(~region+cover+bl+dwc+visit      ~region+cover+bl+gs+dwc, red.umf),

  "p(method+cover+bl+dens+visit)      psi(region+cover+bl+gs + dwc)" =
occu(~method+cover+bl+dens+visit     ~region+cover+bl+gs+dwc, red.umf),

  "p(method+cover+bl+dwc+visit)       psi(region+cover+bl+gs + dwc)" =
occu(~method+cover+bl+dwc+visit      ~region+cover+bl+gs+dwc, red.umf),


  #six terms

  "p(region+method+cover+bl+dens+visit)psi(region+cover+bl+gs + dwc)" =
occu(~region+method+cover+bl+dens+visit~region+cover+bl+gs+dwc, red.umf),

  "p(region+method+cover+bl+dwc+visit) psi(region+cover+bl+gs + dwc)" =
occu(~region+method+cover+bl+dwc+visit ~region+cover+bl+gs+dwc, red.umf)

)

red.p.fl <- fitList(fits=red.p.mods)

red.p.ms <- modSel(red.p.fl)

red.p.ms


### CONCLUSIONS from step 1:

###    - 'region+method+dwc+visit' is aic best p model

###    - bl, cover are 'uninformative parameters' (Arnold 2010)




##################################################################################

## Stage two: compare occupancy models using a aic-best detection structure


red.psi.mods <- list(#~p ~psi


  ##single term

  "p(1)                          psi(1)"      = occu(~1                    ~1,     red.umf),

  "p(region+method+dwc+visit)          psi(1)"      = occu(~region+method+dwc+visit  ~1,     red.umf),
```

```
  "p(region+method+dwc+visit)          psi(region)"  = occu(~region+method+dwc+visit  ~region,
red.umf),
  "p(region+method+dwc+visit)          psi(dens)"    = occu(~region+method+dwc+visit  ~dens,
red.umf),
  "p(region+method+dwc+visit)          psi(dwc)"     = occu(~region+method+dwc+visit  ~dwc,
red.umf),
  "p(region+method+dwc+visit)          psi(cover)"   = occu(~region+method+dwc+visit  ~cover,
red.umf),
  "p(region+method+dwc+visit)          psi(bl)"      = occu(~region+method+dwc+visit  ~bl,     red.umf),
  "p(region+method+dwc+visit)          psi(gs)"      = occu(~region+method+dwc+visit  ~gs,
red.umf),


  ##two term inc *interactions
  "p(region+method+dwc+visit)          psi(region+dens)"  = occu(~region+method+dwc+visit
~region+dens,  red.umf),
  "p(region+method+dwc+visit)          psi(region*dens)"  = occu(~region+method+dwc+visit
~region*dens,  red.umf), #prob
  "p(region+method+dwc+visit)          psi(region+dwc)"   = occu(~region+method+dwc+visit
~region+dwc,  red.umf),
  "p(region+method+dwc+visit)          psi(region*dwc)"   = occu(~region+method+dwc+visit
~region*dwc,  red.umf), #prob
  "p(region+method+dwc+visit)          psi(region+bl)"    = occu(~region+method+dwc+visit
~region+bl,     red.umf),
  "p(region+method+dwc+visit)          psi(region*bl)"    = occu(~region+method+dwc+visit
~region*cover,    red.umf),
  "p(region+method+dwc+visit)          psi(region+cover)" = occu(~region+method+dwc+visit
~region+cover,    red.umf),
  "p(region+method+dwc+visit)          psi(region*cover)" = occu(~region+method+dwc+visit
~region*cover,    red.umf),
  "p(region+method+dwc+visit)          psi(region+gs)"    = occu(~region+method+dwc+visit
~region+gs,   red.umf),
  "p(region+method+dwc+visit)          psi(bl+gs)"        = occu(~region+method+dwc+visit
~bl+gs,   red.umf),
  "p(region+method+dwc+visit)          psi(bl*gs)"        = occu(~region+method+dwc+visit
~bl*gs,   red.umf),
  "p(region+method+dwc+visit)          psi(bl+cover)"     = occu(~region+method+dwc+visit
~bl+cover,    red.umf),
```

"p(region+method+dwc+visit)   psi(bl+dens)"   = occu(~region+method+dwc+visit ~bl+dens, red.umf),

"p(region+method+dwc+visit)   psi(bl+dwc)"   = occu(~region+method+dwc+visit ~bl+dwc, red.umf),

"p(region+method+dwc+visit)   psi(dwc*bl)"   = occu(~region+method+dwc+visit ~dwc*bl, red.umf),

"p(region+method+dwc+visit)   psi(dens*bl)"   = occu(~region+method+dwc+visit ~dens*bl, red.umf),

"p(region+method+dwc+visit)   psi(cover+gs)"   = occu(~region+method+dwc+visit ~cover+gs, red.umf),

"p(region+method+dwc+visit)   psi(cover*gs)"   = occu(~region+method+dwc+visit ~cover*gs, red.umf),

"p(region+method+dwc+visit)   psi(cover+dens)" = occu(~region+method+dwc+visit ~cover+dens, red.umf),

"p(region+method+dwc+visit)   psi(cover+dwc)"   = occu(~region+method+dwc+visit ~cover+dwc, red.umf),

"p(region+method+dwc+visit)   psi(cover*dens)" = occu(~region+method+dwc+visit ~cover*dens, red.umf),

"p(region+method+dwc+visit)   psi(cover*dwc)"   = occu(~region+method+dwc+visit ~cover*dwc, red.umf),

"p(region+method+dwc+visit)   psi(gs+dens)"   = occu(~region+method+dwc+visit ~gs+dens, red.umf),

"p(region+method+dwc+visit)   psi(gs+dwc)"   = occu(~region+method+dwc+visit ~gs+dwc, red.umf),

"p(region+method+dwc+visit)   psi(gs*dens)"   = occu(~region+method+dwc+visit ~gs*dens, red.umf),

"p(region+method+dwc+visit)   psi(gs*dwc)"   = occu(~region+method+dwc+visit ~gs*dwc, red.umf),

##three term w/o interactions

"p(region+method+dwc+visit)   psi(region+bl+cover)"= occu(~region+method+dwc+visit ~region+bl+cover,   red.umf),

"p(region+method+dwc+visit)   psi(region+bl+dens)" = occu(~region+method+dwc+visit ~region+bl+dens, red.umf),

"p(region+method+dwc+visit)   psi(region+bl+dwc)" = occu(~region+method+dwc+visit ~region+bl+dwc, red.umf),

"p(region+method+dwc+visit) Psi(region+bl+gs)" = occu(~region+method+dwc+visit ~region+bl+gs, red.umf),

"p(region+method+dwc+visit) psi(region+cover+dens)"= occu(~region+method+dwc+visit ~region+cover+dens, red.umf),

"p(region+method+dwc+visit) psi(region+cover+dwc)"= occu(~region+method+dwc+visit ~region+cover+dwc, red.umf),

"p(region+method+dwc+visit) psi(region+cover+gs)"= occu(~region+method+dwc+visit ~region+cover+gs, red.umf),

"p(region+method+dwc+visit) psi(region+dens+gs)" = occu(~region+method+dwc+visit ~region+dens+gs, red.umf),

"p(region+method+dwc+visit) psi(region+dwc+gs)" = occu(~region+method+dwc+visit ~region+dwc+gs, red.umf),

"p(region+method+dwc+visit) psi(bl+cover+dens)" = occu(~region+method+dwc+visit ~bl+cover+dens, red.umf),

"p(region+method+dwc+visit) psi(bl+cover+dwc)" = occu(~region+method+dwc+visit ~bl+cover+dwc, red.umf),

"p(region+method+dwc+visit) psi(bl+cover+gs)" = occu(~region+method+dwc+visit ~bl+cover+gs, red.umf),

"p(region+method+dwc+visit) psi(bl+dens+gs)" = occu(~region+method+dwc+visit ~bl+dens+gs, red.umf),

"p(region+method+dwc+visit) psi(bl+dwc+gs)" = occu(~region+method+dwc+visit ~bl+dwc+gs, red.umf),

"p(region+method+dwc+visit) psi(cover+dens+gs)" = occu(~region+method+dwc+visit ~cover+dens+gs, red.umf),

"p(region+method+dwc+visit) psi(cover+dwc+gs)" = occu(~region+method+dwc+visit ~cover+dwc+gs, red.umf),

##four term w/o interactions

"p(region+method+dwc+visit) psi(region+bl+cover+dens)" = occu(~region+method+dwc+visit ~region+bl+cover+dens, red.umf),

"p(region+method+dwc+visit) psi(region+bl+cover+dwc)" =occu(~region+method+dwc+visit ~region+bl+cover+dwc, red.umf),

"p(region+method+dwc+visit) psi(region+bl+cover+gs)" =occu(~region+method+dwc+visit ~region+bl+cover+gs, red.umf),

"p(region+method+dwc+visit) psi(bl+cover+dwc+gs)" =occu(~region+method+dwc+visit ~bl+cover+dwc+gs, red.umf),

"p(region+method+dwc+visit) psi(bl+cover+dens+gs)" =occu(~region+method+dwc+visit ~bl+cover+dens+gs, red.umf),

"p(region+method+dwc+visit) psi(region+bl+gs+dens)" =occu(~region+method+dwc+visit ~region+bl+gs+dens, red.umf),

"p(region+method+dwc+visit) psi(region+bl+gs+dwc)" =occu(~region+method+dwc+visit ~region+bl+gs+dwc, red.umf),

##five term w/o interactions

"p(region+method+dwc+visit) psi(region+bl+cover+gs+dens)" =occu(~region+method+dwc+visit ~region+bl+cover+gs+dens, red.umf),

"p(region+method+dwc+visit) psi(region+bl+cover+gs+dwc)" =occu(~region+method+dwc+visit ~region+bl+cover+gs+dwc, red.umf),

##three term with interactions

"p(region+method+dwc+visit) psi(region*dwc+cover)" =occu(~region+method+dwc+visit ~region*dwc+cover, red.umf),

"p(region+method+dwc+visit) psi(region*dwc+bl)" =occu(~region+method+dwc+visit ~region*dwc+bl, red.umf),

"p(region+method+dwc+visit) psi(region*dwc+gs)" =occu(~region+method+dwc+visit ~region*dwc+gs, red.umf),

"p(region+method+dwc+visit) psi(region*dens+cover)" =occu(~region+method+dwc+visit ~region*dens+cover, red.umf),

"p(region+method+dwc+visit) psi(region*dens+bl)" =occu(~region+method+dwc+visit ~region*dens+bl, red.umf),

"p(region+method+dwc+visit) psi(region*dens+gs)" =occu(~region+method+dwc+visit ~region*dens+gs, red.umf),

"p(region+method+dwc+visit) psi(region*cover+dwc)" =occu(~region+method+dwc+visit ~region*cover+dwc, red.umf),

"p(region+method+dwc+visit) psi(region*cover+dens)" =occu(~region+method+dwc+visit ~region*cover+dens, red.umf),

"p(region+method+dwc+visit) psi(region*cover+bl)" =occu(~region+method+dwc+visit ~region*cover+bl, red.umf),

"p(region+method+dwc+visit) psi(region*cover+gs)" =occu(~region+method+dwc+visit ~region*cover+gs, red.umf),

"p(region+method+dwc+visit) psi(region*bl+dwc)" =occu(~region+method+dwc+visit ~region*bl+dwc, red.umf),

```
  "p(region+method+dwc+visit)     psi(region*bl+dens)"     =occu(~region+method+dwc+visit
~region*bl+dens, red.umf),

  "p(region+method+dwc+visit)     psi(region*bl+cover)"    =occu(~region+method+dwc+visit
~region*bl+cover, red.umf),

  "p(region+method+dwc+visit)     psi(region*bl+gs)"       =occu(~region+method+dwc+visit
~region*bl+gs, red.umf),

  "p(region+method+dwc+visit)     psi(dwc*bl+region)"          =occu(~region+method+dwc+visit
~dwc*bl+region, red.umf),

  "p(region+method+dwc+visit)     psi(dwc*bl+cover)"           =occu(~region+method+dwc+visit
~dwc*bl+cover, red.umf),


  "p(region+method+dwc+visit)     psi(dens*bl+region)"         =occu(~region+method+dwc+visit
~dens*bl+region, red.umf),

  "p(region+method+dwc+visit)     psi(dens*bl+cover)"          =occu(~region+method+dwc+visit
~dens*bl+cover, red.umf),


  "p(region+method+dwc+visit)     psi(dwc*cover+region)"       =occu(~region+method+dwc+visit
~dwc*cover+region, red.umf),

  "p(region+method+dwc+visit)     psi(dwc*cover+bl)"           =occu(~region+method+dwc+visit
~dwc*cover+bl, red.umf),

  "p(region+method+dwc+visit)     psi(dens*cover+region)"      =occu(~region+method+dwc+visit
~dens*cover+region, red.umf),

  "p(region+method+dwc+visit)     psi(dens*cover+bl)"          =occu(~region+method+dwc+visit
~dens*cover+bl, red.umf),

  "p(region+method+dwc+visit)     psi(dwc*cover+gs)"           =occu(~region+method+dwc+visit
~dwc*cover+gs, red.umf),

  "p(region+method+dwc+visit)     psi(dens*cover+gs)"          =occu(~region+method+dwc+visit
~dens*cover+gs, red.umf),

  "p(region+method+dwc+visit)     psi(gs*bl+region)"           =occu(~region+method+dwc+visit
~gs*bl+region, red.umf),

  "p(region+method+dwc+visit      psi(gs*bl+cover)"            =occu(~region+method+dwc+visit
~gs*bl+cover, red.umf),

  "p(region+method+dwc+visit)     psi(gs*bl+dwc)"              =occu(~region+method+dwc+visit
~gs*bl+dwc, red.umf),

  "p(region+method+dwc+visit)     psi(gs*bl+dens)"             =occu(~region+method+dwc+visit
~gs*bl+dens, red.umf),
```

```
  "P(region+method+dwc+visit)     psi(dwc*gs+region)"        =occu(~region+method+dwc+visit
~dwc*gs+region, red.umf),

  "p(region+method+dwc+visit)     psi(dwc*gs+cover)"         =occu(~region+method+dwc+visit
~dwc*gs+cover, red.umf),


  "P(region+method+dwc+visit)     psi(dens*gs+region)"        =occu(~region+method+dwc+visit
~dens*gs+region, red.umf),

  "p(region+method+dwc+visit)     psi(dens*gs+cover)"         =occu(~region+method+dwc+visit
~dens*gs+cover, red.umf),


  "p(region+method+dwc+visit)     psi(gs*cover+region)"    =occu(~region+method+dwc+visit
~gs*cover+region, red.umf),


  "p(region+method+dwc+visit)     psi(gs*cover+dwc)"      =occu(~region+method+dwc+visit
~gs*cover+dwc, red.umf),

  "p(region+method+dwc+visit)     psi(gs*cover+dens)"     =occu(~region+method+dwc+visit
~gs*cover+dens, red.umf),


  ##four term inc interactions:

  "p(region+method+dwc+visit)     psi(region*dwc+cover+bl)"   =occu(~region+method+dwc+visit
~region*dwc+cover+bl, red.umf),

  "p(region+method+dwc+visit)     psi(region*dwc+cover+gs)"   =occu(~region+method+dwc+visit
~region*dwc+cover+gs, red.umf),

  "p(region+method+dwc+visit)     psi(region*dwc+bl+gs)"      =occu(~region+method+dwc+visit
~region*dwc+bl+gs, red.umf),

  "p(region+method+dwc+visit)     psi(region*dens+cover+bl)"  =occu(~region+method+dwc+visit
~region*dens+cover+bl, red.umf),

  "p(region+method+dwc+visit)     psi(region*dens+cover+gs)"  =occu(~region+method+dwc+visit
~region*dens+cover+gs, red.umf),

  "p(region+method+dwc+visit)     psi(region*dens+bl+gs)"     =occu(~region+method+dwc+visit
~region*dens+bl+gs, red.umf),

  "p(region+method+dwc+visit)     psi(region*cover+dwc+bl)"   =occu(~region+method+dwc+visit
~region*cover+dwc+bl, red.umf),

  "p(region+method+dwc+visit)     psi(region*cover+dwc+gs)"   =occu(~region+method+dwc+visit
~region*cover+dwc+gs, red.umf),

  "p(region+method+dwc+visit)     psi(region*cover+dens+bl)"  =occu(~region+method+dwc+visit
~region*cover+dens+bl, red.umf),
```

```
  "p(region+method+dwc+visit)      psi(region*cover+dens+gs)"  =occu(~region+method+dwc+visit
~region*cover+dens+gs, red.umf),
  "p(region+method+dwc+visit)      psi(region*cover+bl+gs)"    =occu(~region+method+dwc+visit
~region*cover+bl+gs, red.umf),
  "p(region+method+dwc+visit)      psi(region*bl+dwc+cover)"   =occu(~region+method+dwc+visit
~region*bl+dwc+cover, red.umf),


  "p(region+method+dwc+visit)      psi(region*bl+dens+cover)"  =occu(~region+method+dwc+visit
~region*bl+dens+cover, red.umf),


  "p(region+method+dwc+visit)      psi(dwc*bl+region+cover)"   =occu(~region+method+dwc+visit
~dwc*bl+region+cover, red.umf),


  "p(region+method+dwc+visit)      psi(dens*bl+region+cover)"  =occu(~region+method+dwc+visit
~dens*bl+region+cover, red.umf),


  "p(region+method+dwc+visit)      psi(dwc*gs+region+cover)"   =occu(~region+method+dwc+visit
~dwc*gs+region+cover, red.umf),



  "p(region+method+dwc+visit)      psi(dens*gs+region+cover)"  =occu(~region+method+dwc+visit
~dens*gs+region+cover, red.umf),


  "p(region+method+dwc+visit)      psi(dwc*cover+region+bl)"   =occu(~region+method+dwc+visit
~dwc*cover+region+bl, red.umf),
  "p(region+method+dwc+visit)      psi(dwc*cover+region+gs"    =occu(~region+method+dwc+visit
~dwc*cover+region+gs, red.umf),
  "p(region+method+dwc+visit)      psi(dwc*cover+bl+gs)"       =occu(~region+method+dwc+visit
~dwc*cover+bl+gs, red.umf),
  "p(region+method+dwc+visit)      psi(dens*cover+region+bl)"  =occu(~region+method+dwc+visit
~dens*cover+region+bl, red.umf),
  "p(region+method+dwc+visit)      psi(dens*cover+region+gs)"  =occu(~region+method+dwc+visit
~dens*cover+region+gs, red.umf),
  "p(region+method+dwc+visit)      psi(dens*cover+bl+gs)"      =occu(~region+method+dwc+visit
~dens*cover+bl+gs, red.umf),
```

```
  "p(region+method+dwc+visit)      psi(gs*cover+region+dwc)"   =occu(~region+method+dwc+visit
~gs*cover+region+dwc, red.umf),

  "p(region+method+dwc+visit)      psi(gs*cover+region+dens)"  =occu(~region+method+dwc+visit
~gs*cover+region+dens, red.umf),


  "p(region+method+dwc+visit)      psi(gs*bl+region+cover)"    =occu(~region+method+dwc+visit
~gs*bl+region+cover, red.umf),

  "p(region+method+dwc+visit)      psi(gs*bl+region+dwc)"      =occu(~region+method+dwc+visit
~gs*bl+region+dwc, red.umf),

  "p(region+method+dwc+visit)      psi(gs*bl+region+dens)"     =occu(~region+method+dwc+visit
~gs*bl+region+dwc, red.umf),

  "p(region+method+dwc+visit)      psi(gs*bl+cover+dwc)"       =occu(~region+method+dwc+visit
~gs*bl+cover+dwc, red.umf),

  "p(region+method+dwc+visit)      psi(gs*bl+cover+dens)"      =occu(~region+method+dwc+visit
~gs*bl+cover+dens, red.umf),


  #five term with interactions

  "p(region+method+dwc+visit)      psi(region*dwc+cover+bl+gs)"  =occu(~region+method+dwc+visit
~region*dwc+cover+bl+gs, red.umf),

  "p(region+method+dwc+visit)      psi(region*dens+cover+bl+gs)"  =occu(~region+method+dwc+visit
~region*dens+cover+bl+gs, red.umf),

  "p(region+method+dwc+visit)      psi(region*cover+dwc+bl+gs)"   =occu(~region+method+dwc+visit
~region*cover+dwc+bl+gs, red.umf),

  "p(region+method+dwc+visit)      psi(region*cover+dens+bl+gs)"  =occu(~region+method+dwc+visit
~region*cover+dens+bl+gs, red.umf),


  "p(region+method+dwc+visit)      psi(gs*bl+region+cover+dwc)"   =occu(~region+method+dwc+visit
~gs*bl+region+cover+dwc, red.umf),

  "p(region+method+dwc+visit)      psi(gs*bl+region+cover+dens)"  =occu(~region+method+dwc+visit
~gs*bl+region+cover+dens, red.umf),

  "p(region+method+dwc+visit)      psi(dwc*cover+region+gs+bl)"   =occu(~region+method+dwc+visit
~dwc*cover+region+gs+bl, red.umf),

  "p(region+method+dwc+visit)      psi(dens*cover+region+gs+bl)"  =occu(~region+method+dwc+visit
~dens*cover+region+gs+bl, red.umf)

)
```

```
#find (and then remove) models that did not converge:

if(1==2){

  red.psi.fl <- fitList(fits=red.psi.mods)

  red.psi.ms <- modSel(red.psi.fl)

  red.psi.ms

  for(i in 1:132){

    print(i)

    modSel(fitList(fits=red.psi.mods[1:i]))

  }

}


noncon <- c(14,16,67,69,92,104,119)


red.psi.fl <- fitList(fits=red.psi.mods[-noncon])

red.psi.ms <- modSel(red.psi.fl)

red.psi.ms


### CONCLUSIONS from step 2:

###    - bl+cover+dwc basically the aic-top, and kind of clear top model

###    - the dwc:cover and dwc:bl interactions and gs are 'uninformative parameters' (Arnold 2010)

###    - some model uncertainty though, so model do average predictions



######################################################

# Coeficient table


coef.tab <- red.psi.ms@Full

write.csv(coef.tab, file="red_coef_tab.csv")
```

```
#################################################
# Model average predictions

# occupancy ~ dwc [holding bl, cover, dens, and gs @ ???]

#################################################
# model averaged predictions: RS ~ dwc

nn <- 500

region <- rep(c("BO","CS","HI"), each=nn)

dwc <- c(with(red[red$region %in% "BO",],seq(min(dwc),max(dwc),length=nn)),
         with(red[red$region %in% "CS",],seq(min(dwc),max(dwc),length=nn)),
         with(red[red$region %in% "HI",],seq(min(dwc),max(dwc),length=nn)))

bl <- c(with(red[red$region %in% "BO",],seq(min(bl),max(bl),length=nn)),
        with(red[red$region %in% "CS",],seq(min(bl),max(bl),length=nn)),
        with(red[red$region %in% "HI",],seq(min(bl),max(bl),length=nn)))

dens <- c(with(red[red$region %in% "BO",],seq(min(dens),max(dens),length=nn)),
          with(red[red$region %in% "CS",],seq(min(dens),max(dens),length=nn)),
          with(red[red$region %in% "HI",],seq(min(dens),max(dens),length=nn)))

cover <- c(with(red[red$region %in% "BO",],seq(min(cover),max(cover),length=nn)),
           with(red[red$region %in% "CS",],seq(min(cover),max(cover),length=nn)),
           with(red[red$region %in% "HI",],seq(min(cover),max(cover),length=nn)))

gs <- c(with(red[red$region %in% "BO",],seq(min(gs),max(gs),length=nn)),
        with(red[red$region %in% "CS",],seq(min(gs),max(gs),length=nn)),
        with(red[red$region %in% "HI",],seq(min(gs),max(gs),length=nn)))
```

```
for(j in 1:2){

mid_metric <- c("mean","median")[j]

BO.val <- apply(red[red$region%in%"BO",c("bl","cover","dens","dwc","gs")],2,mid_metric, na.rm=T)
CS.val <- apply(red[red$region%in%"CS",c("bl","cover","dens","dwc","gs")],2,mid_metric, na.rm=T)
HI.val <- apply(red[red$region%in%"HI",c("bl","cover","dens","dwc","gs")],2,mid_metric, na.rm=T)

bl.mu    <- c(rep(BO.val[1], nn), rep(CS.val[1], nn), rep(HI.val[1], nn))
cover.mu <- c(rep(BO.val[2], nn), rep(CS.val[2], nn), rep(HI.val[2], nn))
dens.mu  <- c(rep(BO.val[3], nn), rep(CS.val[3], nn), rep(HI.val[3], nn))
dwc.mu   <- c(rep(BO.val[4], nn), rep(CS.val[4], nn), rep(HI.val[4], nn))
gs.mu    <- c(rep(BO.val[5], nn), rep(CS.val[5], nn), rep(HI.val[5], nn))

new.df.r <- data.frame(region = region,
                  bl     = bl.mu,      #mean bl
                  dens   = dens.mu,    #mean dens
                  cover  = cover.mu,   #mean cover
                  dwc    = dwc,        #vary dwc
                  gs     = gs.mu)      #mean gs

pred.dwc.r <- predict(red.psi.fl, type="state", newdata = new.df.r)

# Plot - can decide on type and resolution here too:
for(i in 1:3){
  if(i == 1) pdf(paste0("red_by_dwc.se.",mid_metric,".pdf"), height=6,     width=6)
  if(i == 2) png(paste0("red_by_dwc.se.",mid_metric,".png"), height = 500, width = 500, pointsize = 
16, res=72)
  if(i == 3) tiff(paste0("red_by_dwc.se.",mid_metric,".tif"),height = 500, width = 500, pointsize = 16, 
res=72)
```

```
  plot(new.df.r$dwc, new.df.r$dwc, ylim=c(0,1), type="n", xlab="Pine Marten Connectivity",
       ylab="Occupancy", bty="l", las=1)

  polygon(c(new.df.r$dwc[1:nn],rev(new.df.r$dwc[1:nn])),
          c(pred.dwc.r[1:nn,1]+pred.dwc.r[1:nn,2],rev(pred.dwc.r[1:nn,1]-pred.dwc.r[1:nn,2])),
          border=F,col=adjustcolor("darkblue",0.5))

  polygon(c(new.df.r$dwc[(nn+1):(nn*2)],rev(new.df.r$dwc[(nn+1):(nn*2)])),
          c(pred.dwc.r[(nn+1):(nn*2),1]+pred.dwc.r[(nn+1):(nn*2),2],
            rev(pred.dwc.r[(nn+1):(nn*2),1]-pred.dwc.r[(nn+1):(nn*2),2])),
          border=F,col=adjustcolor("darkgreen",0.5))

  polygon(c(new.df.r$dwc[(nn+nn+1):(nn*3)],rev(new.df.r$dwc[(nn+nn+1):(nn*3)])),
          c(pred.dwc.r[(nn+nn+1):(nn*3),1]+pred.dwc.r[(nn+nn+1):(nn*3),2],
            rev(pred.dwc.r[(nn+nn+1):(nn*3),1]-pred.dwc.r[(nn+nn+1):(nn*3),2])),
          border=F,col=adjustcolor("darkred",0.5))

  lines(new.df.r$dwc[1:nn],pred.dwc.r[1:nn,1],lwd=2)
  lines(new.df.r$dwc[(nn+1):(nn*2)],pred.dwc.r[(nn+1):(nn*2),1],lwd=2)
  lines(new.df.r$dwc[(nn+nn+1):(nn*3)],pred.dwc.r[(nn+nn+1):(nn*3),1],lwd=2)

  legend("topleft",c("Borders","Central","Highlands"),pch=15, bty="n",
         col=adjustcolor(c("darkblue","darkgreen","darkred")),cex=1.2)
  dev.off()
}
}
```
