# Supplementary material for: The enemy of my enemy is my friend: native pine marten recovery reverses the decline of the red squirrel by suppressing grey squirrel populations
Source: Proc Biol Sci. 2018 Mar 7;285(1874):20172603. doi: 10.1098/rspb.2017.2603 (PMC5879625; doi:10.1098/rspb.2017.2603)
Supplement: Table S5 from The enemy of my enemy is my friend: Native pine marten recovery reverses the decline of the red squirrel by suppressing grey squirrel populations [file rspb20172603supp5.pdf]

Table S5. The number of locations at which each species was detected according to method (HAIR or CAMERA) in the Central (CS), Borders (BO) and Highlands (HI) regions of Scotland (as proportion of available devices in parentheses), n = number of sites

		red squirrel		grey squirrel		pine marten	
region	n	HAIR	CAMERA	HAIR	CAMERA	HAIR	CAMERA
CS	107	32 (0.30)	16 (0.39)	19 (0.18)	9 (0.22)	76 (0.71)	25 (0.61)
BO	80	7 (0.09)	4 (0.17)	32 (0.40)	5 (0.21)	43 (0.54)	12 (0.5)
HI	36	20 (0.56)	6 (0.75)	-	-	36 (1)	8 (1)
