# Supplementary material for: The enemy of my enemy is my friend: native pine marten recovery reverses the decline of the red squirrel by suppressing grey squirrel populations
Source: Proc Biol Sci. 2018 Mar 7;285(1874):20172603. doi: 10.1098/rspb.2017.2603 (PMC5879625; doi:10.1098/rspb.2017.2603)
Supplement: Table S6 from The enemy of my enemy is my friend: Native pine marten recovery reverses the decline of the red squirrel by suppressing grey squirrel populations [file rspb20172603supp6.pdf]

Table S6 Model selection for grey squirrels and corresponding model specific β coefficient estimates for covariates determining grey squirrel occupancy (Ψ) and probability of detection (*p*). K = no. of parameters, ΔAIC = difference in AIC value from top model, AIC Wt = AIC weight, c Wt = cumulative AIC weight.

Model						Ψ Coefficients						
p	Ψ	K	ΔAIC	AIC Wt	c Wt	Intercept	DWC	DENS	COVER	BL	REGION(CS)	*Interaction
p(REGION+DWC+VISIT)	Ψ (REGION*BL+DWC)	11	0.00	0.55	0.55	0.75 ± 0.52	-1.32 ± 0.34	-	-	4.70 ± 2.16	-0.97 ± 0.62	-5.02 ± 2.26
p(REGION+DWC+VISIT)	Ψ (REGION*BL+DWC+COVER)	12	2.00	0.20	0.75	0.83 ± 1.76	-1.32 ± 0.34	-	-0.09 ± 1.79	4.69 ± 2.18	-0.97 ± 0.62	-5.02 ± 2.26
p(REGION+DWC+VISIT)	Ψ (REGION+DWC)	9	4.94	0.05	0.80	1.11 ± 0.48	-1.30 ± 0.31	-	-	-	-1.57 ± 0.44	-
p(REGION+DWC+VISIT)	Ψ (REGION*DWC)	10	5.71	0.03	0.83	0.81 ± 0.49	-1.04 ± 0.36	-	-	-	-1.01 ± 0.63	-0.69 ± 0.64
p(REGION+DWC+VISIT)	Ψ (REGION+BL+DWC)	10	6.00	0.03	0.86	0.97 ± 0.49	-1.24 ± 0.31	-	-	0.54 ± 0.57	-1.85 ± 0.54	-
p(REGION+DWC+VISIT)	Ψ (REGION+COVER+DWC)	10	6.56	0.02	0.88	1.97 ± 1.54	-1.26 ± 0.31	-	-0.94 ± 1.56	-	-1.62 ± 0.46	-
p(REGION+DWC+VISIT)	Ψ (REGION*DWC+BL)	11	6.70	0.02	0.90	0.64 ± 0.51	-0.96 ± 0.37	-	-	0.56 ± 0.57	-1.28 ± 0.70	-0.70 ± 0.63
p(REGION+DWC+VISIT)	Ψ (REGION*DWC+COVER)	11	7.36	0.01	0.91	1.57 ± 1.42	-0.99 ± 0.37	-	-0.86 ± 1.47	-	-1.03 ± 0.65	-0.69 ± 0.64
p(REGION+DWC+VISIT)	Ψ (REGION*COVER+DWC)	11	7.63	0.01	0.93	9.31 ± 7.58	-1.48 ± 0.39	-	-8.11 ± 7.48	-	-9.57 ± 7.81	8.01 ± 7.87
p(REGION+DWC+VISIT)	Ψ (DWC*BL+REGION)	11	7.82	0.01	0.94	0.82 ± 0.57	-1.12 ± 0.41	-	-	0.74 ± 0.74	-1.81 ± 0.55	-0.29 ± 0.66
p(REGION+DWC+VISIT)	Ψ (REGION*BL+DENS)	11	7.84	0.01	0.95	-0.50 ± 0.30	-	-8.67 ± 4.30	-	3.96 ± 2.14	-0.62 ± 0.57	-4.00 ± 2.23
p(REGION+DWC+VISIT)	Ψ (REGION+BL+COVER+DWC)	11	7.93	0.01	0.96	1.39 ± 1.65	-1.23 ± 0.31	-	-0.45 ± 1.65	0.48 ± 0.61	-1.84 ± 0.55	-
p(REGION+DWC+VISIT)	Ψ (1)	7	38.06	-	0.00	-	-	-	-	-	-	-
p (1)	Ψ (1)	2	87.73	-	0.00	-	-	-	-	-	-	-
						p Coefficients						
						Intercept	REGION (CS)	DWC	VISIT 2	VISIT 3	VISIT 4	VISIT 5
p(REGION+DWC+VISIT)	Ψ (REGION*BL+DWC)					-2.62 ± 0.48	1.61 ± 0.38	0.74 ± 0.26	1.37 ± 0.46	1.71 ± 0.47	3.10 ± 0.53	-
