# Supplementary material for: The enemy of my enemy is my friend: native pine marten recovery reverses the decline of the red squirrel by suppressing grey squirrel populations
Source: Proc Biol Sci. 2018 Mar 7;285(1874):20172603. doi: 10.1098/rspb.2017.2603 (PMC5879625; doi:10.1098/rspb.2017.2603)
Supplement: Table S7 from The enemy of my enemy is my friend: Native pine marten recovery reverses the decline of the red squirrel by suppressing grey squirrel populations [file rspb20172603supp7.pdf]

Table S7 Model selection for red squirrels and corresponding model specific β coefficient estimates for covariates determining red squirrel occupancy (Ψ) and probability of detection (*p*). K = no. of parameters, ΔAIC = difference in AIC value from top model, AIC Wt = AIC weight, c Wt = cumulative AIC weight.

Model						Ψ Coefficients							
p	Ψ	K	ΔAIC	AIC Wt	c Wt	Intercept	DWC	COVER	BL	GS	REGION (CS)	REGION (HI)	*Interaction
p (REGION+METHOD+DWC+VISIT)	Ψ (BL+COVER+DWC)	13	0.00	0.17	0.17	-3.68 ± 1.45	0.81 ± 0.32	3.21 ± 1.46	-1.18 ± 0.47	-	-	-	-
p (REGION+METHOD+DWC+VISIT)	Ψ (DWC*COVER+BL)	14	0.52	0.13	0.30	-6.41 ± 2.99	2.67 ± 1.71	6.19 ± 3.15	-1.12 ± 0.47	-	-	-	-2.11 ± 1.85
p (REGION+METHOD+DWC+VISIT)	Ψ (DWC*BL+COVER)	14	0.99	0.10	0.40	-3.31 ± 1.47	0.58 ± 0.37	3.09 ± 1.45	-1.88 ± 0.86	-	-	-	0.66 ± 0.67
p (REGION+METHOD+DWC+VISIT)	Ψ (BL+COVER+DWC+GS)	14	1.95	0.06	0.46	-3.77 ± 1.50	0.85 ± 0.36	3.23 ± 1.46	-1.18 ± 0.47	0.15 ± 0.68	-	-	-
p (REGION+METHOD+DWC+VISIT)	Ψ (GS*COVER+DWC)	14	2.09	0.06	0.52	-3.43 ± 1.48	0.87 ± 0.32	2.25 ± 1.49	-	-23.90 ± 14.35	-	-	25.10 ± 14.74
p (REGION+METHOD+DWC+VISIT)	Ψ (DWC*COVER+BL+GS)	15	2.44	0.05	0.57	-6.53 ± 3.02	2.74 ± 1.73	6.24 ± 3.15	-1.12 ± 0.47	0.20 ± 0.69	-	-	-2.13 ± 1.86
p (REGION+METHOD+DWC+VISIT)	Ψ (GS*BL+COVER+DWC)	15	3.47	0.03	0.60	-3.78 ± 1.49	0.85 ± 0.36	3.15 ± 1.45	-1.02 ± 0.52	0.63 ± 0.97	-	-	-0.98 ± 1.44
p (REGION+METHOD+DWC+VISIT)	Ψ (BL+DWC)	12	3.62	0.03	0.63	-0.73 ± 0.43	0.79 ± 0.30	-	-1.31 ± 0.45	-	-	-	-
p (REGION+METHOD+DWC+VISIT)	Ψ (REGION+BL+COVER+DWC)	15	3.74	0.03	0.65	-3.79 ± 1.52	0.77 ± 0.46	3.03 ± 1.48	-1.28 ± 0.53	-	0.42 ± 0.92	0.27 ± 1.27	-
p (REGION+METHOD+DWC+VISIT)	Ψ (DWC*BL+REGION+COVER)	16	4.23	0.02	0.67	- 3.59 ± 1.51	0.25 ± 0.51	3.02 ± 1.47	-2.14 ± 0.89	-	0.70 ± 0.74	1.02 ± 1.07	0.89 ± 0.71
p (REGION+METHOD+DWC+VISIT)	Ψ (DWC*BL)	13	4.25	0.02	0.69	-0.44 ± 0.49	0.54 ± 0.35	-	-2.07 ± 0.82	-	-	-	0.73 ± 0.64
p (REGION+METHOD+DWC+VISIT)	Ψ (DWC*COVER+REGION+BL)	16	4.25	0.02	0.71	-6.71 ± 3.18	2.62 ± 1.72	6.16 ± 3.27	-1.19 ± 0.53	-	0.48 ± 0.88	0.47 ± 1.18	-2.14 ± 1.87
p (REGION+METHOD+DWC+VISIT)	Ψ (DWC*COVER)	13	4.36	0.02	0.73	-8.29 ± 3.15	3.30 ± 1.74	7.45 ± 3.34	-	-	-	-	-
p (REGION+METHOD+DWC+VISIT)	Ψ (REGION*COVER+DWC+BL)	17	4.38	0.02	0.75	-21.27 ± 21.85	0.84 ± 0.47	21.29 ± 22.38	-1.19 ± 0.55	-	16.73 ± 21.89	19.42 ± 21.88	-17.12 ± 22.47 (CS),<br>-20.37 ± 22.51 (HI)
p (REGION+METHOD+DWC+VISIT)	Ψ (COVER+DWC)	12	4.51	0.02	0.77	-4.88 ± 1.44	0.98 ± 0.31	3.71 ± 1.49	-	-	-	-	-
p (REGION+METHOD+DWC+VISIT)	Ψ (1)	10	24.54	-	-	-0.51 ± 0.17	-	-	-	-	-	-	-
p (1)	Ψ (1)	2	181.26	-	-	-0.92 ± 0.16	-	-	-	-	-	-	-

			p Coefficients							
		Intercept	REGION (CS)	REGION (HI)	METHOD (HAIR)	DWC	VISIT 2	VISIT 3	VISIT 4	VISIT 5
p (REGION+METHOD+DWC+VISIT)	Ψ (BL+COVER+DWC)	2.00 ± 1.27	6.22 ± 0.92	7.12 ± 1.09	-6.59 ± 1.48	-2.64 ± 0.42	1.40 ± 0.54	2.86 ± 0.58	2.91 ± 0.58	3.59 ± 0.75
