# Supplementary material for: The enemy of my enemy is my friend: native pine marten recovery reverses the decline of the red squirrel by suppressing grey squirrel populations
Source: Proc Biol Sci. 2018 Mar 7;285(1874):20172603. doi: 10.1098/rspb.2017.2603 (PMC5879625; doi:10.1098/rspb.2017.2603)
Supplement: File S8 from The enemy of my enemy is my friend: Native pine marten recovery reverses the decline of the red squirrel by suppressing grey squirrel populations [file rspb20172603supp8.pdf]

```
###### SCR_MODEL_FIT

#need to install the oSCR package!

library(oSCR)
library(rgdal)
library(rgeos)
library(raster)
library(smoothie)
#Need to place the 'enc.csv' ans 'traps.csv' files in a folder somewhere
#Execute the following line, and when prompted, point to the folder you put the files in:

############################################
#Step 1: creat the encounter history data:

enc <- read.csv("pm_scr/pm_scr_data/enc.csv",h=T)
traps <- read.csv("pm_scr/pm_scr_data/traps.csv",h=T)

head(enc)
head(traps)

enc$trapID   <- factor(enc$trapID,levels=unique(traps$trapID))
enc$occasion <- factor(enc$occasion,levels=1:5)
enc$session  <- factor(enc$session,levels=unique(traps$region))
enc$id       <- factor(enc$id,levels=unique(enc$id))

capls   <- list()
trapls  <- list()
indCovs <- list()
trapOp  <- list()
```

```
for(i in 1:length(unique(enc$session))){ #area is session - multi session model
  ii      <- unique(enc$session)[i]
  tmp.enc <- enc[enc$session %in% ii,]
  tmp.trp <- droplevels(traps[traps$region %in% ii,])
  tmp.enc$trapID   <- factor(tmp.enc$trapID, levels=unique(tmp.trp$trapID))
  tmp.enc$id       <- droplevels(tmp.enc$id)
  tmp.enc$occasion <- droplevels(tmp.enc$occasion)
  tmp.y <- with(tmp.enc, tapply(id,list(id,trapID,occasion),length))
  tmp.y[is.na(tmp.y)] <- 0
  tmp.sex <- ifelse(table(tmp.enc$id,tmp.enc$sex)[,1]>0,0,1)
  to.mat  <- with(tmp.trp,cbind(ifelse(tapply(o1,trapID,sum)>0,1,0),
                          ifelse(tapply(o2,trapID,sum)>0,1,0),
                          ifelse(tapply(o3,trapID,sum)>0,1,0),
                          ifelse(tapply(o4,trapID,sum)>0,1,0),
                          ifelse(tapply(o5,trapID,sum)>0,1,0)))
  capls[[i]]   <- tmp.y
  trapls[[i]]  <- tmp.trp[,c("X","Y")]/1000
  indCovs[[i]] <- data.frame(sex=tmp.sex)
  trapOp[[i]]  <- to.mat
}

sf <- make.scrFrame(caphist       = capls,      #encounterd ind x trap x occassion
                    traps         = trapls,     #trap locations
                    indCovs       = indCovs,    #sex data
                    trapOperation = trapOp)     #trap operation



##################################
#Step 1: create the state space:


#ss is the mask.
```

```
#it is a list containing 3 dataframes, 1 for each region
#each dataframe has an x and a y coordinate.
#density covariates are defined at each point.

buff <- 4
ress <- 0.1
calc.se <- TRUE

# need forest only habitat
forest <- raster("GIS//forestry_25m")

ss <- list()

for(i in 1:3){
  tmp.sp       <- SpatialPoints(sf$traps[[i]][,c("X","Y")]*1000)
  tmp.buff     <- gBuffer(tmp.sp, width = buff*1000)
  tmp.r        <- crop(forest, extent(tmp.buff))
  values(tmp.r) <- ifelse(values(tmp.r)>0,1,0)
  tmp.r        <- aggregate(mask(tmp.r,tmp.buff),fact=4,fun=mean)
  ss[[i]]      <- as.data.frame(coordinates(tmp.r)[!is.na(values(tmp.r)) & values(tmp.r)>0,]/1000)
  colnames(ss[[i]]) <- c("X","Y")
}


###################################
#Analysis:
#possible models:
#  p0  ~ 1 / session / sex / sex+session
#  sig ~ 1 / session / sex / sex+session
```

```
#  D   ~ 1 / session

mods <- make.mods(density   = c(~1,~session),
              detection = c(~1, ~sex, ~session, ~sex+session),
              sigma     = c(~1, ~sex, ~session, ~sex+session),
              cost      = c(~1))

modList <- my.parfit(mods = mods,
                data = list(sf=sf,ss=ss,cs=NULL),
                ncores = 3)
load("models.RData")

fl <- fitList.oSCR(out, rename=T)
ms <- modSel.oSCR(fl)
save.image(file = "pm_scr/pm_scr_output/allMods.RData")
```

```
if(1==2){
modList <- list(
  m0  =oSCR.fit(model=list(D~1,       p0~1,           sig~1),             sf,ss,trimS=buff),
  m1  =oSCR.fit(model=list(D~1,       p0~session,     sig~1),             sf,ss,trimS=buff),
  m2  =oSCR.fit(model=list(D~1,       p0~sex,         sig~1),             sf,ss,trimS=buff),
  m3  =oSCR.fit(model=list(D~1,       p0~session+sex, sig~1),             sf,ss,trimS=buff),
  m4  =oSCR.fit(model=list(D~1,       p0~1,           sig~sex),           sf,ss,trimS=buff),
  m5  =oSCR.fit(model=list(D~1,       p0~session,     sig~sex),           sf,ss,trimS=buff),
  m6  =oSCR.fit(model=list(D~1,       p0~sex,         sig~sex),           sf,ss,trimS=buff),
  m7  =oSCR.fit(model=list(D~1,       p0~session+sex, sig~sex),           sf,ss,trimS=buff),
  m8  =oSCR.fit(model=list(D~1,       p0~1,           sig~session),       sf,ss,trimS=buff),
  m9  =oSCR.fit(model=list(D~1,       p0~session,     sig~session),       sf,ss,trimS=buff),
  m10 =oSCR.fit(model=list(D~1,       p0~sex,         sig~session),       sf,ss,trimS=buff),
  m11 =oSCR.fit(model=list(D~1,       p0~session+sex, sig~session),       sf,ss,trimS=buff),
  m12 =oSCR.fit(model=list(D~1,       p0~1,           sig~session + sex), sf,ss,trimS=buff),
  m13 =oSCR.fit(model=list(D~1,       p0~session,     sig~session + sex), sf,ss,trimS=buff),
  m14 =oSCR.fit(model=list(D~1,       p0~sex,         sig~session + sex), sf,ss,trimS=buff),
  m15 =oSCR.fit(model=list(D~1,       p0~session+sex, sig~session + sex), sf,ss,trimS=buff),
  m16 =oSCR.fit(model=list(D~session, p0~1,           sig~1),             sf,ss,trimS=buff),
  m17 =oSCR.fit(model=list(D~session, p0~session,     sig~1),             sf,ss,trimS=buff),
  m18 =oSCR.fit(model=list(D~session, p0~sex,         sig~1),             sf,ss,trimS=buff),
  m19 =oSCR.fit(model=list(D~session, p0~session+sex, sig~1),             sf,ss,trimS=buff),
  m20 =oSCR.fit(model=list(D~session, p0~1,           sig~sex),           sf,ss,trimS=buff),
  m21 =oSCR.fit(model=list(D~session, p0~session,     sig~sex),           sf,ss,trimS=buff),
  m22 =oSCR.fit(model=list(D~session, p0~sex,         sig~sex),           sf,ss,trimS=buff),
  m23 =oSCR.fit(model=list(D~session, p0~session+sex, sig~sex),           sf,ss,trimS=buff),
  m24 =oSCR.fit(model=list(D~session, p0~1,           sig~session),       sf,ss,trimS=buff),
  m25 =oSCR.fit(model=list(D~session, p0~session,     sig~session),       sf,ss,trimS=buff),
```

```
  m26 =oSCR.fit(model=list(D~session, p0~sex,         sig~session),       sf,ss,trimS=buff),
  m27 =oSCR.fit(model=list(D~session, p0~session+sex, sig~session),       sf,ss,trimS=buff),
  m28 =oSCR.fit(model=list(D~session, p0~1,           sig~session + sex), sf,ss,trimS=buff),
  m29 =oSCR.fit(model=list(D~session, p0~session,     sig~session + sex), sf,ss,trimS=buff),
  m30 =oSCR.fit(model=list(D~session, p0~sex,         sig~session + sex), sf,ss,trimS=buff),
  m31 =oSCR.fit(model=list(D~session, p0~session+sex, sig~session + sex), sf,ss,trimS=buff)
)
}
```

```
my.parfit <- function (mods, data, ncores = 3){
  library(doParallel)
  sf <- data$sf
  ss <- data$ss
  cs <- data$cs
  wrapperX <- function(model.number, mods, sf, ss, cs = NULL) {
    mod <- list(mods[[1]][[model.number]],
                mods[[2]][[model.number]],
                mods[[3]][[model.number]],
                mods[[4]][[model.number]])
    fm <- oSCR.fit(scrFrame = sf,
                   ssDF = ss,
                   costDF = cs,
                   model = mod,
                   trimS = 4,
                   se = TRUE,
                   distmet = "euc",
```

```
                sexmod = "constant")
    save(fm, file = paste("model", model.number, ".RData", sep = ""))
    return(fm)
  }
  nmods <- nrow(mods)
  cl <- makeCluster(ncores)
  registerDoParallel(cl)
  out <- foreach(i = 1:nmods) %dopar% {
    library(oSCR)
    tmp <- wrapperX(i, mods, sf, ss)
    return(tmp)
  }
  stopCluster(cl)
  save("out", file = "models.RData")
  tmp <- list()
  for (i in 1:length(out)) {
    tmp[[i]] <- out[[i]]
    tmp[[i]]$call$model <- list("list", paste(mods[i, 1]),
                              paste(mods[i, 2]), paste(mods[i, 3]), paste(mods[i,
                                                                          4]))
  }
  out <- fitList.oSCR(tmp, rename = TRUE)
  return(out)
}


#########################################################
                          ####  SCR_SURFACE_CREATION


library(oSCR)
library(smoothie)
```

```
library(SDMTools)
library(raster)

load("pm_scr/pm_scr_output/allMods.RData")



load("models.RData")
fl <- fitList.oSCR(out, rename = T)
ms <- modSel.oSCR(fl)
if(!"realizedDensity.Rdata" %in% list.files("pm_scr/pm_scr_output/")){
  ## Model averaged realized density
  dens.s1 <- NULL
  dens.s2 <- NULL
  ord <- order(unlist(sapply(fl,function(x)x$AIC)))
  tmp.wts <- wts <- ms$aic.tab$weight
  wts[ord] <- tmp.wts
  pred <- my.predict(scr.fit = fl[[1]], override.trim = T)
#  pred <- predict.oSCR(scr.fit = fl[[1]], override.trim = T)
  dens.s1 <- pred$r[[1]] * wts[1]
  dens.s2 <- pred$r[[2]] * wts[1]
  dens.s3 <- pred$r[[3]] * wts[1]

  for(i in 2:length(fl)){
    pred <- my.predict(scr.fit = fl[[i]], override.trim = T)
#    pred <- predict.oSCR(scr.fit = fl[[i]], override.trim = T)
    dens.s1 <- pred$r[[1]] + pred$r[[1]] * wts[i]
    dens.s2 <- pred$r[[2]] + pred$r[[2]] * wts[i]
    dens.s3 <- pred$r[[3]] + pred$r[[3]] * wts[i]
  }

  realizedDensity <- list(dens.s1,dens.s2,dens.s3)
```

```
  save(realizedDensity,file="pm_scr/pm_scr_output/realizedDensity.Rdata")
}else{
  load("pm_scr/pm_scr_output/realizedDensity.Rdata")
}


## smoothed model averaged realized density

if(!"aveDensity.Rdata" %in% list.files("pm_scr/pm_scr_output/")){
  #pixel=0.1
  #HR diameter ~ 0.250
  r = 1.5
  #sig = 0.125/2

  pix.res <- 0.1
  sig <- (0.125 / 2) / pix.res

  #make the smooths:
  rds1 <- realizedDensity[[1]]
  rds2 <- realizedDensity[[2]]
  rds3 <- realizedDensity[[3]]

  values(rds1) <- kernel2dsmooth(as.matrix(realizedDensity[[1]]),
                                 kernel.type="disk",
                                 r = r)
  values(rds2) <- kernel2dsmooth(as.matrix(realizedDensity[[2]]),
                                 kernel.type="disk",
                                 r = r)
  values(rds3) <- kernel2dsmooth(as.matrix(realizedDensity[[3]]),
                                 kernel.type="disk",
                                 r = r)
```

```
  aveDensity <- list(rds1, rds2, rds3)
  save(aveDensity,file="pm_scr/pm_scr_output/aveDensity.Rdata")
}else{
  load("pm_scr/pm_scr_output/aveDensity.Rdata")
}

#Expected Density

if(!"expectedDensity.Rdata" %in% list.files("pm_scr/pm_scr_output/")){
  mav <- ma.coef(modSel.oSCR(fl))
  d0 <- mav$Estimate[mav$Parameter %in% "d0.(Intercept)"]
  d1 <- mav$Estimate[mav$Parameter %in% "d.beta.session2"]
  d2 <- mav$Estimate[mav$Parameter %in% "d.beta.session3"]

  ed1 <- exp(d0)
  ed2 <- exp(d0+d1)
  ed3 <- exp(d0+d2)

  rde1 <- realizedDensity[[1]]
  rde2 <- realizedDensity[[2]]
  rde3 <- realizedDensity[[3]]
  values(rde1) <- values(rde1) * 0 + ed1
  values(rde2) <- values(rde2) * 0 + ed2
  values(rde3) <- values(rde3) * 0 + ed3
  expectedDensity <- list(rde1, rde2, rde3)
  save(expectedDensity,file="pm_scr/pm_scr_output/expectedDensity.Rdata")
}else{
  load("pm_scr/pm_scr_output/expectedDensity.Rdata")
}
```

```
#DWC

if(!"dwc.Rdata" %in% list.files("pm_scr/pm_scr_output/")){
  dwc <- list(realizedDensity[[1]],
              realizedDensity[[2]],
              realizedDensity[[3]])

  mav <- ma.coef(modSel.oSCR(fl))
  psi <- plogis(mav$Estimate[mav$Parameter %in% "psi.constant"])
  s0  <- mav$Estimate[mav$Parameter       %in% "sig.(Intercept)"]
  s1  <- mav$Estimate[mav$Parameter       %in% "sig.session2"]
  s2  <- mav$Estimate[mav$Parameter       %in% "sig.session3"]
  sM  <- mav$Estimate[mav$Parameter       %in% "sig.sexmale"]

  sf  <- exp(c(s0,s0+s1,s0+s2))
  sm  <- exp(c(s0+sM,s0+s1+sM,s0+s2+sM))

  for(i in 1:3){
    tmp.dwc <- rep(NA,ncell(dwc[[i]]))
    for(j in 1:ncell(dwc[[i]])){
#     if(!is.na(values(dwc[[i]])[j])){
        tmp.d <- e2dist(coordinates(dwc[[i]])[j,,drop=F],
                        coordinates(dwc[[i]]))
        tmp.val <- values(dwc[[i]])
        tmp.val[is.na(tmp.val)] <- 0
        tmp.dwc[j] <- sum(tmp.val *
                          exp(-(tmp.d^2 / (2*sf[i]^2))) *
                          (1-psi) +
                          tmp.val *
                          exp(-(tmp.d^2 / (2*sm[i]^2))) *
                          psi)
```

```
#     }
      print(paste0(i,": ",round(100*j/ncell(dwc[[i]]),2),"%"))
    }
    values(dwc[[i]]) <- tmp.dwc
  }
  save(dwc,file="pm_scr/pm_scr_output/dwc.Rdata")
}else{
  load("pm_scr/pm_scr_output/dwc.Rdata")
}

## smoothed model averaged dwc

if(!"aveDWC.Rdata" %in% list.files("pm_scr/pm_scr_output/")){
  #pixel=0.1
  #HR diameter ~ 0.250
  #HR r = 0.125
  #sig = 0.125/2
  r=1.5
  pix.res <- 0.1
  sig <- (0.125 / 2) / pix.res

  #make the smooths:
  dwcs1 <- dwc[[1]]
  dwcs2 <- dwc[[2]]
  dwcs3 <- dwc[[3]]

  values(dwcs1) <- kernel2dsmooth(as.matrix(dwc[[1]]),
                                  kernel.type="disk",
                                  r = r)
  values(dwcs2) <- kernel2dsmooth(as.matrix(dwc[[2]]),
                                  kernel.type="disk",
```

```
                          r = r)
  values(dwcs3) <- kernel2dsmooth(as.matrix(dwc[[3]]),
                          kernel.type="disk",
                          r = r)
  aveDWC <- list(dwcs1, dwcs2, dwcs3)
  save(aveDWC,file="pm_scr/pm_scr_output/aveDWC.Rdata")
}else{
  load("pm_scr/pm_scr_output/aveDWC.Rdata")
}

##################################
# plot density and dwc
clr <- colorRampPalette(c("white","darkblue"))(1000)
sf <- fl[[1]]$scrFrame
ss <- fl[[1]]$ssDF

brk.d <- seq(min(c(values(aveDensity[[1]]),values(aveDensity[[2]]),values(aveDensity[[3]]))),
          max(c(values(aveDensity[[1]]),values(aveDensity[[2]]),values(aveDensity[[3]]))),
          length=1000)
brk.dwc <- seq(min(c(values(aveDWC[[1]]),values(aveDWC[[2]]),values(aveDWC[[3]]))),
            max(c(values(aveDWC[[1]]),values(aveDWC[[2]]),values(aveDWC[[3]]))),
            length=1000)
legend.xy <- cbind(x=c(339,341,341,339), y=c(650,650,660,660))

#pdf("martenSurfaces.pdf",height=6,width=9)
tiff("martenSurfaces.tif",height=1000,width=1500,pointsize = 30, res=72)
par(mfrow=c(2,3),oma=c(0,3,0,0),mar=c(1,3,2,1))
wot <- aveDensity
plot(wot[[1]],breaks=brk.d,axes=F,box=TRUE,legend.width=2,col=clr,main="Borders",colNA="white",
    legend=F)
mtext(side=2,"Smoothed Density", line = 1)
```

```
legend.gradient(legend.xy, cols = clr, title=" ", c(round(min(brk.d),3),round(max(brk.d),3)))

points(sf$traps[[1]],pch=3,cex=0.5)

plot(wot[[2]],breaks=brk.d,axes=F,box=TRUE,legend.width=2,col=clr,main="Highland",colNA="white
",

    legend=F)

points(sf$traps[[2]],pch=3,cex=0.5)

plot(wot[[3]],breaks=brk.d,axes=F,box=TRUE,legend.width=2,col=clr,main="Loch
Lomond",colNA="white",

    legend=F)

points(sf$traps[[3]],pch=3,cex=0.5)


wot <- aveDWC

plot(wot[[1]],breaks=brk.dwc,axes=F,box=TRUE,legend.width=2,col=clr,main=" ",colNA="white",

    legend=F)

mtext(side=2,"Smoothed DWC")

legend.gradient(legend.xy, cols = clr, title=" ", c(round(min(brk.dwc),3),round(max(brk.dwc),3)))

points(sf$traps[[1]],pch=3,cex=0.5)

plot(wot[[2]],breaks=brk.dwc,axes=F,box=TRUE,legend.width=2,col=clr,main=" ",colNA="white",

    legend=F)

points(sf$traps[[2]],pch=3,cex=0.5)

plot(wot[[3]],breaks=brk.dwc,axes=F,box=TRUE,legend.width=2,col=clr,main=" ",colNA="white",

    legend=F)

points(sf$traps[[3]],pch=3,cex=0.5)

dev.off()



pdf("martenSurfaces.pdf",height=3,width=9)

#tiff("martenSurfacesMS.tif",height=500,width=1500,pointsize = 30, res=72)

par(mfrow=c(1,3),oma=c(0,3,0,0),mar=c(3,3,2,1))

wot <- aveDWC

plot(wot[[1]],breaks=brk.dwc,axes=F,box=TRUE,legend.width=2,col=clr,main="Borders",colNA="whit
e",
```

```
    legend=F)
mtext(side=2,"DWC", line = 2)
legend.gradient(legend.xy, cols = clr, title=" ", c(round(min(brk.dwc),3),round(max(brk.dwc),3)))
points(sf$traps[[1]],pch=16,cex=0.5)
arrows(min(coordinates(wot[[1]])[,1])+2,
      max(coordinates(wot[[1]])[,2]),
      min(coordinates(wot[[1]])[,1])+7,
      max(coordinates(wot[[1]])[,2]), lwd=2, length = 0)
text(min(coordinates(wot[[1]])[,1])+4,max(coordinates(wot[[1]])[,2])-2,"5 km")


plot(wot[[3]],breaks=brk.dwc,axes=F,box=TRUE,legend.width=2,col=clr,main="Central",colNA="white",
    legend=F)
points(sf$traps[[3]],pch=16,cex=0.5)
arrows(min(coordinates(wot[[3]])[,1])+2,
      max(coordinates(wot[[3]])[,2])-2,
      min(coordinates(wot[[3]])[,1])+7,
      max(coordinates(wot[[3]])[,2])-2, lwd=2, length = 0)
text(min(coordinates(wot[[3]])[,1])+4,max(coordinates(wot[[3]])[,2])-4,"5 km")

plot(wot[[2]],breaks=brk.dwc,axes=F,box=TRUE,legend.width=2,col=clr,main="Highland",colNA="white",
    legend=F)
points(sf$traps[[2]],pch=16,cex=0.5)
arrows(min(coordinates(wot[[2]])[,1]),
      max(coordinates(wot[[2]])[,2])-1.5,
      min(coordinates(wot[[2]])[,1])+5,
      max(coordinates(wot[[2]])[,2])-1.5, lwd=2, length = 0)
text(min(coordinates(wot[[2]])[,1])+1,max(coordinates(wot[[2]])[,2])-2.5,"5 km")
dev.off()
```

```
my.predict <-
function (scr.fit, scrFrame = NULL, ssDF = NULL, costDF = NULL,
        rsfDF = NULL, override.trim = FALSE)
{
  library(sp)
  library(raster)
  mles <- scr.fit$rawOutput$estimate
  call <- scr.fit$call
  oSCR.fit2 <- oSCR.fit
  call.fix <- names(call)[!names(call) %in% c("", "scrFrame",
                                        "ssDF", "costDF", "rsfDF")]
  if (override.trim)
    call.fix <- call.fix[call.fix != "trimS"]
  if (length(call.fix > 0)) {
    for (i in 1:length(call.fix)) {
      formals(oSCR.fit2)[[call.fix[i]]] <- call[[call.fix[i]]]
    }
  }
  if (is.null(scrFrame)) {
    sf <- scr.fit$scrFrame
  }
  else {
    sf <- scrFrame
  }
  if (is.null(ssDF)) {
    ss <- scr.fit$ssDF
  }
  else {
    ss <- ssDF
  }
```

```
if (is.null(costDF)) {
  cs <- scr.fit$costDF
}
else {
  cs <- costDF
}
if (is.null(rsfDF)) {
  rs <- scr.fit$rsfDF
}
else {
  rs <- ssDF
}
out <- oSCR.fit2(model = lapply(scr.fit$model,as.formula), scrFrame = sf, ssDF = ss, costDF = cs,
                rsfDF = rs, start.vals = mles, predict = TRUE)
nsess <- length(out$preds)
r <- list()
total <- list()
pbar <- list()
for (s in 1:nsess) {
  nguys <- dim(out$preds[[s]])[1]
  Nhat <- sum(out$ss.bits[[s]][, "d.s"])
  n0 <- out$ss.bits[[s]][, "d.s"] * out$ss.bits[[s]][, "lik.cond"]
  tmp <- SpatialPoints(out$ssDF[[s]][, c("X", "Y")])
  tmp <- try(sp::points2grid(tmp))
  if (class(tmp) == "try-error") {
    cat("Cannot rasterize state-space", fill = TRUE)
    pbar[[s]] <- cbind(out$ssDF[[s]][, c("X", "Y")],
                  pbar = (1 - out$ss.bits[[s]][, "lik.cond"]))
  }
  else {
    pbar[[s]] <- rasterFromXYZ(cbind(out$ssDF[[s]][,
```

```
                                    c("X", "Y")], pbar = (1 - out$ss.bits[[s]][,
                                                          "lik.cond"])))
    }
    cat("Nhat: ", sum(n0) + nguys - 1, fill = TRUE)
    out$preds[[s]][nguys, ] <- n0
    total[[s]] <- apply(out$preds[[s]], 2, sum)
    cat("sum of predictions: ", sum(total[[s]]), fill = TRUE)
    if (class(tmp) == "try-error") {
      r[[s]] <- cbind(out$ssDF[[s]][, c("X", "Y")], total[[s]])
    }
    else {
      r[[s]] <- rasterFromXYZ(cbind(out$ssDF[[s]][, c("X",
                                    "Y")], total[[s]]))
    }
  }
  return(list(r = r, ssN = total, preds = out$preds, ssDF = out$ssDF,
         pbar = pbar))
}
```
